# Comparative Pathogenomics Reveals Horizontally Acquired Novel Virulence Genes in Fungi Infecting Cereal Hosts

**DOI:** 10.1371/journal.ppat.1002952

**Published:** 2012-09-27

**Authors:** Donald M. Gardiner, Megan C. McDonald, Lorenzo Covarelli, Peter S. Solomon, Anca G. Rusu, Mhairi Marshall, Kemal Kazan, Sukumar Chakraborty, Bruce A. McDonald, John M. Manners

**Affiliations:** 1 Commonwealth Scientific and Industrial Research Organization (CSIRO) Plant Industry, Queensland Bioscience Precinct, Brisbane, Queensland, Australia; 2 Plant Pathology, Institute of Integrative Biology, ETH Zurich, Zürich, Switzerland; 3 Dipartimento di Scienze Agrarie e Ambientali, Faculty of Agriculture, University of Perugia, Forgo XX Giugno, Perugia, Italy; 4 Research School of Biology, College of Medicine, Biology and Environment, The Australian National University, Canberra, Australia; 5 Queensland Facility for Advanced Bioinformatics, The University of Queensland, Brisbane, Queensland, Australia; Carnegie Mellon University, United States of America

## Abstract

Comparative analyses of pathogen genomes provide new insights into how pathogens have evolved common and divergent virulence strategies to invade related plant species. Fusarium crown and root rots are important diseases of wheat and barley world-wide. In Australia, these diseases are primarily caused by the fungal pathogen *Fusarium pseudograminearum*. Comparative genomic analyses showed that the *F. pseudograminearum* genome encodes proteins that are present in other fungal pathogens of cereals but absent in non-cereal pathogens. In some cases, these cereal pathogen specific genes were also found in bacteria associated with plants. Phylogenetic analysis of selected *F. pseudograminearum* genes supported the hypothesis of horizontal gene transfer into diverse cereal pathogens. Two horizontally acquired genes with no previously known role in fungal pathogenesis were studied functionally via gene knockout methods and shown to significantly affect virulence of *F. pseudograminearum* on the cereal hosts wheat and barley. Our results indicate using comparative genomics to identify genes specific to pathogens of related hosts reveals novel virulence genes and illustrates the importance of horizontal gene transfer in the evolution of plant infecting fungal pathogens.

## Author Summary

Cereals are our most important staple crops and are subject to attack from a diverse range of fungal pathogens. A major goal of molecular plant pathology research is to understand how pathogens infect plants to allow the development of durable plant protection measures. Comparing the genomes of different pathogens of cereals and contrasting them to non-cereal pathogen genomes allows for the identification of genes important for pathogenicity toward these important crops. In this study, we sequenced the genome of the wheat and barley pathogen *F. pseudograminearum* responsible for crown and root-rot diseases, and compared it to those from a broad range of previously sequenced fungal genomes from cereal and non-cereal pathogens. These analyses revealed that the *F. pseudograminearum* genome contains a number of genes only found in fungi pathogenic on cereals. Some of these genes appear to have been horizontally acquired from other fungi and, in some cases, from plant associated bacteria. The functions of two of these genes were tested by creating strains that lacked the genes. Both genes had important roles in causing disease on cereals. This work has important implications for our understanding of pathogen specialization during the evolution of fungal pathogens infecting cereal crops.

## Introduction

Crop losses due to fungal pathogens represent one of the most serious threats to global food production. Staple cereal crops such as wheat, barley, rice and maize are subject to attack from a diverse array of fungal pathogens including biotrophs such as rust fungi that feed on living cells and necrotrophs such as *Fusarium* pathogens that kill host cells to obtain nutrients. Many *Fusarium* pathogens not only reduce crop yields but also produce mycotoxins that are harmful to humans and livestock when consumed in food and feed. A better understanding of the infection strategies used by these pathogens would help develop novel plant protection strategies. Comparative analysis of pathogen genomes offers a new and powerful approach to identify common and divergent virulence strategies as well as evolutionary history of pathogen lineages.

Shared virulence strategies may be used by different fungi to invade specific plant hosts. Presumably in many cases, the existence of common virulence strategies in different pathogen species may be explained by conservation of virulence gene function through vertical inheritance and/or exposure to common host defensive selection forces during pathogenesis on the same or related hosts. However, in some instances, horizontal gene transfer events have been identified in fungal pathogens and subsequently shown to have roles in pathogenicity [1]–[3]. A striking example of a locus-specific horizontal gene transfer event emerged from the sequencing of the wheat pathogen *Phaeosphaeria nodorum* (anamorph *Stagonospora nodorum*) genome, where a gene encoding a host-specific protein toxin (ToxA) was identified by homology to a known toxin from another wheat pathogen *Pyrenophora tritici-repentis*. Functional analyses demonstrated that ToxA was necessary for virulence in both pathogens [1]. It was proposed that transfer of *ToxA* from *P. nodorum to P. tritici-repentis* resulted in the emergence of the tan spot disease of wheat caused by *P. tritici-repentis* in the 1930s [1], [4]. In another example, genome analysis of the tomato vascular wilt pathogen *F. oxysporum* f. sp. *lycopersici* revealed the presence of several supernumerary chromosomes. Non-sexual transfer of one of these chromosomes to a non-virulent and genetically diverged recipient strain was shown to be sufficient to confer virulence on tomato [2]. Recently, association genomics has been used to identify the fungal effector Ave1 (for Avirulence on Ve1 tomato) in *Verticillium dahliae*. *Ave1* homologs were shown to be present in diverse plant pathogenic fungi and important for virulence in at least one fungal species and one plant pathogenic bacterium [5]. In addition, Ave1 had strong homologies to plant proteins, suggesting that a cross-kingdom gene transfer event from plant to fungi may have occurred [5].

Ancient horizontally acquired virulence genes that have been retained because of their selective advantage may have more subtle sequence homologies and therefore are harder to detect [6], [7]. Such genes are best identified by gene phylogenies where a gene associates with a clade of sequences from unrelated species and relationships are incongruent with established ancestries [7]. For example, Klosterman et al [3] found the presence of a glucosyltransferase encoding gene in the genomes of three different wilt causing fungi, *F. oxysporum* f. sp. *lycopersici*, *V. dahliae*, and *V. albo-atrum* as well as an insect pathogen *Metarhizium anisopliae* that was otherwise found only in bacteria. These authors proposed that the acquisition of this gene predates the *Verticillium* spp. split and probably occurred independently in *F. oxysporum* f. sp. *lycopersici* [3]. Using targeted gene disruption approaches, the glucosyltransferase was shown to be important for virulence in *V. dahliae* toward tobacco (*Nicotiana benthamiana*) but not lettuce (*Lactuca sativa*). Similarly, it was recently suggested that the ability to synthesize auxin in members of both the *Fusarium* and *Colletotrichum* genera has probably resulted from horizontal transfer of auxin biosynthetic genes from bacteria [8].

Recent advances in genomics are revolutionizing the analysis of fungal species, which are particularly suited to analysis by the current generation of DNA sequencing technologies due to their relatively small genomes and in many cases minimal repetitive DNA contents. The *de novo* detection of shared virulence strategies without *a priori* information on the roles of shared genes in pathogen virulence offers an exciting possibility of uncovering new insights into the pathogenesis-related processes. The work of Klosterman et al [3] is one of the few examples where nothing was known about the role of the identified genes in pathogen virulence prior to being identified through comparative genomics. Therefore, it is reasonable to expect that further unbiased comparative genomic analyses will uncover examples of shared virulence genes or niche specialization genes in these pathogens, and ultimately provide insights into the co-evolution of virulence/niche specialization functions and the mechanisms of plant defense.

In this paper, we report the sequencing, assembly and annotation of the genome of *Fusarium pseudograminearum* (Aoki and O'Donnell) using Illumina sequencing and a comparative genomics analysis of its gene content. In many parts of the world, *Fusarium pseudograminearum* is the principal cause of Fusarium crown rot (FCR) of wheat and barley. FCR is significant in arid cereal-growing regions worldwide including South Africa [9], Northern Africa [10], the Middle East [11], Europe [12] and Australia [13] as well as the northwest of the United States of America [14]. In Australia, FCR is a chronic problem and among the most economically significant diseases of both wheat and barley [15], [16]. The recent increase in incidence of FCR is ascribed to the increased use of conservation farming practices such as zero tillage and stubble retention. These practices permit the survival of fungal inoculum on residual plant matter across planting seasons. *Fusarium* root-rot is a related cereal disease caused by *F. pseudograminearum* as well as other fusaria [17], [18]. *F. pseudograminearum* was initially distinguished from *F. graminearum* by host tissue preferences [19] and on the basis of molecular data was formally recognized as a separate species [20], [21]. Multi-locus sequence analysis of diverse isolates has shown that *F. pseudograminearum* is a single phylogenetic species globally [22] in contrast to the *F. graminearum* species complex, which shows geographical structure [23], [24]. In addition *F. pseudograminearum* is heterothallic whilst *F. graminearum* has a homothallic mating system. Whilst *F. pseudograminearum*, *F. graminearum* and *F. culmorum* are present throughout the Australian wheat growing regions and can all cause FCR, *F. pseudograminearum* is the species most commonly recovered from plants showing FCR symptoms [25], [26]. Field surveys in Australia have revealed that *F. graminearum* is the most frequently isolated species from wheat plants showing Fusarium head blight (FHB) symptoms [26], although *F. pseudograminearum* can also cause FHB disease. These observations suggest that *F. pseudograminearum*, while a broadly adapted cereal pathogen, may have evolved adaptations for niche specialization or infection processes that favor stem and/or crown infection.

We hypothesized that at least some genes in the *F. pseudograminearum* genome that were either uniquely or predominantly present in other cereal fungal pathogens may have specialized functions related to cereal pathogenesis and niche specialization. Throughout the manuscript the term ‘niche specialization’ is used to encompass other potential aspects of the biology of these fungi for which are poorly understood such saprophytic colonization of dead plant material, non-pathogenic interactions with other plants or potential endophytism of other hosts. A broad comparative genomic analysis indicated that the *F. pseudograminearum* genome contains genes that have strong homology to genes that are unevenly distributed across cereal pathogens while being apparently absent in other fungal genomes. Some of these genes shared by cereal pathogens also encode proteins with significant similarity to those from plant-associated bacteria. This finding is consistent with multiple horizontal acquisition events and indeed phylogenetic analysis of selected genes supported the hypothesis of horizontal gene transfer into diverse cereal pathogens. Functional analysis of two potentially horizontally acquired genes revealed important roles in the virulence of *F. pseudograminearum* on cereal hosts. Our results illustrate an important role for horizontal gene transfer in the evolution of cereal associated fungi.

## Results

### *F. pseudograminearum* Genome Sequence

The genome of *F. pseudograminearum* isolate CS3096 was sequenced using a combination of paired and single-end Illumina reads. *De novo* assembly of these reads resulted in a nuclear genome size of 36.8 Mbp assembled in 656 contigs with 50% of all nucleotides in contigs of 189 Kbp in length or greater (AFNW00000000). The average sequence coverage across these contigs was 179-fold. Compared to many other fungal genome assemblies using next generation sequencing technologies, the *F. pseudograminearum* genome sequence assembly has a relatively high N50 (Table 1). Gene model predictions from three programs were combined to identify 12,448 protein coding gene (see materials and methods).

**Table 1:** Summary of the *F. pseudograminearum* genome sequence assembly in comparison to selected fungal genome sequences obtained by short-read sequencing and the reference genome for *F. graminearum*.

Species	Genome size (Mbp)	Technology	coverage	N50 (kb)	Reference
*F. pseudograminearum*	37	Illumina	179	180	This work
*F. oxysporum* strain 5176	55	454	8×	60	[56]
*Pyrenophora teres f. teres*	42	Illumina	*	26	[77]
*Sordaria macrospora*	39.9	Illumina+454	95†	117	[78]
*Grosmannia clavigera*	30	Illumina	∼100	237	[79]
*Puccinia striiformis*	79	Illumina	50	5	[80]
*Fusarium graminearum*	36.2	Sanger (reference genome)	10	5350	[28]

* not provided.

† 85× coverage with Illumina and 10× with 454.

The repeated sequence content of the *F. pseudograminearum* genome, assessed using the RepeatMasker program, is only 1.6%, which is slightly higher than that of the *F. graminearum* genome (0.7%) assessed using identical parameters, albeit that the sequencing and assembly methodology differed. RepeatMasker recognizes both simple sequence repeats and transposable elements present in RepBase [27]. Although approximately four times as many base pairs were flagged as being derived from Gypsy type Long Terminal Repeat (LTR) elements in the *F. pseudograminearum* genome (26 Kbp) compared to *F. graminearum* (6 Kbp), the difference in repetitive DNA content could mostly be attributed to a higher level of simple repeats and low complexity DNA (1.5% versus 0.41% of the genome, respectively). One high coverage contig in *F. pseudograminearum* encodes a LTR-type retrotransposon with best match to an *F. oxysporum* transposon, probably present in 9–10 copies based on an average coverage of 1689 fold. This transposon matched to sequences in a number of different fungi and also to transposons in both monocot and dicot plants. The contig showed no polymorphism in the sequence read assembly across 5.5 Kbp, suggesting all copies are identical and thus could not be placed in other assembled contigs. Also not included in the overall repeat counts are the contigs that represent rRNA encoding genes. The Illumina sequencing approach used here was unable to resolve these repeats in *F. pseudograminearum* and these currently appear in the assembly as high-coverage contigs.

**Figure 1:**
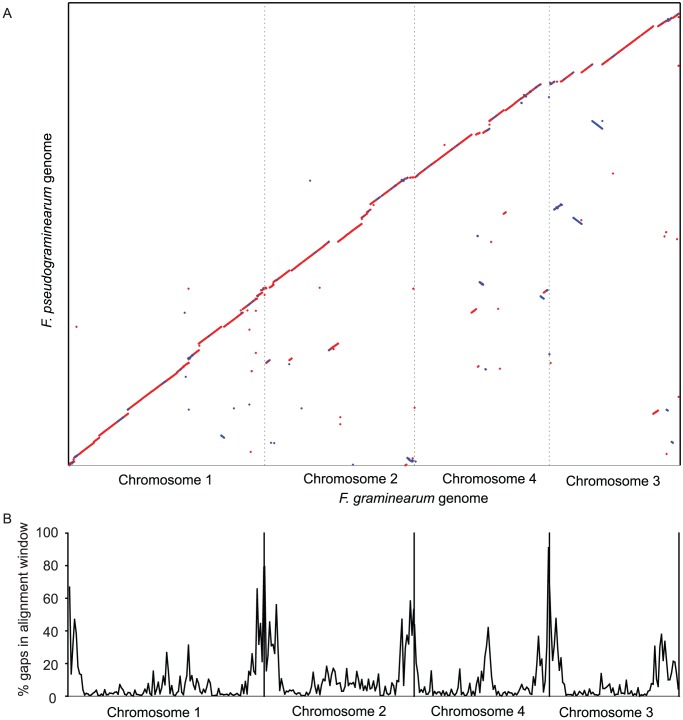
Alignment between *F. graminearum* and *F. pseudograminearum*. *F. graminearum* chromosomes are ordered in decreasing size. (A) Dot-plot representing a whole genome alignment between *F. graminearum* isolate Ph1 and *F. pseudograminearum* isolate CS3096. The alignment was generated with NUCmer, part of the MUMmer 3 comparative sequence analysis package. Sequences were pre-masked for known repetitive elements and simple repeats using RepeatMasker. The dot plot represents the best 1∶1 alignment between the two genomes. Dots closest to the diagonal represent co-linearity between the two genomes. Red represents matches in the forward direction and blue is indicative of inversions in part of the mapped contig relative to the *F. graminearum* genome. (B) Distribution of gaps in the alignment between *F. pseudograminearum* and *F. graminearum*, relative to the *F. graminearum* genome. *F. graminearum* chromosomes were divided into 100 Kbp non-overlapping windows and the unaligned nucleotides in each window summed and expressed as a percentage.

The *F. pseudograminearum* genome sequence assembly was also compared globally to that of *F. graminearum* [28] by aligning the two genomes after masking simple repeat sequences and known fungal repetitive elements. In total, 89.8% of the *F. pseudograminearum* genomic sequence could be aligned to the *F. graminearum* genome at >70% nucleotide identity (Figure 1). An alignment with increased sensitivity was performed using a six frame translations of both genomes enabling an alignment of 94% of the *F. pseudograminearum* genome to that of *F. graminearum*. Thus, at least 6% of the low copy region of the genome (approximately 2.2 Mbp) appears to be completely unique to *F. pseudograminearum*. Very few rearrangements between the *F. pseudograminearum* and *F. graminearum* genomes in the aligned regions were found (Figure 1A). The amount of aligned sequence between the two species decreases towards the ends of the *F. graminearum* chromosomes (Figure 1B) and also in regions previously reported to be undergoing higher rates of genome innovation [28].

### Comparative Genomics of *F. pseudograminearum* and Signatures of Horizontal Gene Transfer into Cereal Pathogens

Genes showing a distribution of homologues limited to cereal pathogens would be both candidates for strong selection, because of a possible involvement in pathogenicity, and for horizontal acquisition. To determine whether the *F. pseudograminearum* predicted gene set contained such candidates, a BLASTmatrix analysis pipeline based on the identification of gene homologues by reciprocal best BLASTp hits was developed using predicted proteomes from the genome sequence of 16 different cereal pathogens and 11 non-pathogens of cereals. The entire BLASTmatrix analysis is presented in Dataset S1 along with the filtered protein sets described below. A numerical summary of the matches is shown in Table S1. In these analyses, 156 predicted *F. pseudograminearum* proteins did not have reciprocal best BLAST hits of any strength in any of the 27 organisms, while 239 predicted proteins had reciprocal best BLASTp matches only in cereal pathogens, with a score of at least 200 bits. In contrast, applying the same filtering criteria to identify proteins that are found in *F. pseudograminearum* and only non-cereal pathogens in this BLASTmatrix analysis identified only 32 proteins, 24 of which were present in the Fusarium lineage.

Amongst the 239 cereal pathogen proteins, 214 had matches within the two other *Fusarium* cereal pathogens (*F. graminearum* and *F. verticillioides*) and not in other species. Amongst the remaining 25 *F. pseudograminearum* proteins, only five had no match in other fusaria while matching other cereal pathogen proteins. Since the genome sampling described herein was limited to 27 fungal genomes, the sets of 239 and 156 proteins were combined and additionally curated using BLASTp against the NCBI nr (non-redundant) database. This process identified a total of 17 proteins with strong BLASTp matches (>200 bits) to bacterial proteins, and six of these 17 proteins were present only in a small number of other fungi (Table 2). The corresponding genes for these proteins are therefore good candidates for having been ancestrally acquired from bacteria. The GC content for each of these six genes was not obviously different to that of surrounding genes, possibly with the exception of FPSE_07765 (see below). Other candidates for cereal pathogen specificity, which to varying degrees showed distributions limited to cereal pathogens, were also identified through the BLASTmatrix filtering. These are therefore also of interest with respect to putative functions in niche specialisation and virulence towards plants. Examples of predicted genes from this latter category suggesting conserved roles in virulence towards cereals, and/or horizontal gene transfer are shown in Table 3. These candidate genes were subjected to more detailed analysis of sequence relationships as described below.

**Table 2:** *Fusarium pseudograminearum* genes that may be of bacterial origin and are candidates for roles in pathogenicity towards plants.

*Fusarium pseudograminearum protein*	*Fusarium graminearum* †	*Fusarium oxysporum f. sp. lycopersici*	*Fusarium oxysporum strain 5176*	*Fusarium verticillioides* †	*Fusarium solani*	*Trichoderma virens*	*Colletotrichum higginsianum*	*Colletotrichum graminicola* †	*Neurospora crassa*	*Magnaporthe poae* †	*Magnaporthe oryzae* †	*Gaeumannomyces graminis var. tritici* †	*Botrytis cinerea*	*Aspergillus nidulans*	*Cochliobolus sativus* †	*Mycosphaerella graminicola* †	*Mycosphaerella fijiensis*	*Cochliobolus heterostrophus C4* †	*Cochliobolus heterostrophus C5* †	*Pyrenophora teres f. teres* †	*Pyrenophora tritici-repentis* †	*Phaeosphaeria nodorum* †	*Saccharomyces cerevisiae*	*Phanerochaete chrysosporium*	*Ustilago maydis* †	*Puccinia triticina* †	*Puccinia graminis var. tritici* †	Bacterial match score	Number of Fungal matches *	Predicted function and best bacterial match species
FPSE_00725	1099															63.2												591	1	*Amidohydrolase. Streptomyces violaceusniger* e-value ∼0
FpAH1																						507						416	1	Amidohydrolase. gamma proteobacterium. E-value 1 e-136
FPSE_07765	1885			1673																								1264	1	Aminotransferase. 75% identical match in a plant endophytic bacterium *Microbacterium testaceum*
FPSE_07775	624			549																							32	361	1	Hydrolase. Best bacterial match is in the human opportunistic pathogen *Segniliparus rugosus* 57% identical, e-value 1e-122
FPSE_11221	577											336																403	1	NAD(P)+ dependent epimerase/hydratase Oxalobacteraceae bacterium 58% identical e-value 1e-136
FPSE_11233	1452			724						601		660			634			635	635	409	631	613						397	6	Possibly a hydrolytic enzyme 38% identical to *Streptomyces hygroscopicus*, e-value of 1e-125

Proteins showing distribution limited to cereal pathogens in a BLASTmatrix analysis were used as queries of the non-redundant protein database at NCBI and those for which specificity to pathogens was still evident and had strong matches in bacterial species, with a limited number of fungal matches were retained. Numbers indicate BLASTp bit scores from the reciprocal best blast hits. The score for the best bacterial blast hit is also shown.

† Cereal pathogen

* Number of fungal matches better than the first bacterial hit in GenBank’s nr.

**Table 3:** Examples of *Fusarium pseudograminearum* genes that may be of fungal origin and are candidates for roles in cereal pathogenicity.

*Fusarium pseudograminearum protein*	*Fusarium graminearum* †	*Fusarium oxysporum f. sp. lycopersici*	*Fusarium oxysporum strain 5176*	*Fusarium verticillioides* †	*Fusarium solani*	*Trichoderma virens*	*Colletotrichum higginsianum*	*Colletotrichum graminicola* †	*Neurospora crassa*	*Magnaporthe poae* †	*Magnaporthe oryzae* †	*Gaeumannomyces graminis var. tritici* †	*Botrytis cinerea*	*Aspergillus nidulans*	*Mycosphaerella graminicola* †	*Mycosphaerella fijiensis*	*Cochliobolus sativus* †	*Cochliobolus heterostrophus C4* †	*Cochliobolus heterostrophus C5* †	*Pyrenophora teres f. teres* †	*Pyrenophora tritici-repentis* †	*Phaeosphaeria nodorum* †	*Saccharomyces cerevisiae*	*Phanerochaete chrysosporium*	*Ustilago maydis* †	*Puccinia triticina* †	*Puccinia graminis var. tritici* †	Putative function
FPSE_02381	299																											Small secreted cysteine rich protein
FPSE_02497	1148			1044				677		632		654																Choline dehydrogenase
FPSE_05718																	357											Transcription factor
FPSE_05719															437													Cytochrome P450 monooxygenase
FPSE_05720																												No reciprocal best blast hits in any fungi in the BLASTmatrix. Encodes an amidase
FPSE_06956	365			254						113	133	98																Small secreted cysteine rich protein
FpDLH1				541				502																				Dienelactone hydrolase
FPSE_10646	274																					55						Killer protein 4 homologues. Distributed in many ascomycetes. Calcium channel inhibitors.

Numbers indicate BLASTp bit scores from the reciprocal best blast hits.

† Cereal pathogen

### Putative Functions and Sequence Relationships of Selected Cereal Pathogen Genes

Amongst the *F. pseudograminearum* genes of potential bacterial origin (Table 2), the intronless gene *FPSE_07765* and its orthologues in *F. graminearum* and *F. verticillioides* encode proteins highly similar to a bacterial protein with both aminotransferase and homoserine kinase domains. FPSE_07765 is 75% identical at the amino acid level across the entire length to a protein from *Microbacterium testaceum*, which is a bacterial endophyte of a variety of plants including the cereals sorghum and maize [29], [30]. Other fungal hits to this sequence in GenBank only align across less than half of the protein and at much lower identities, with the best fungal match in *Trichophyton verrucosum* at only 31% amino acid identity and with only partial query coverage of 44%. Phylogenetic analysis of FPSE_07765 identifies a *Fusarium* sequence clade embedded amongst a range of related bacterial sequences and strongly supports an origin of this gene via horizontal acquisition from bacteria, with retention in the cereal infecting fusaria (Figure S1). The GC content of *FPSE_07765* was 58% compared to the genome average of 51.8±3.9% (±SD) for coding sequences.

*FPSE_11233*, another *F. pseudograminearum* gene with possible horizontal acquisition, encodes a putative secreted hydrolase that has reciprocal best BLAST hits only in cereal pathogens among the 27 selected genomes as well as hits in GenBank to putative pectin- and xylan-hydrolases in bacteria (best match is 38% identical with an e-value of 1e^−125^ to a *Streptomyces hygroscopicus* protein). Further manual database queries showed FPSE_11233-like sequences in the cereal endophyte *Chaetomium globosum* and the *Brassica* pathogen *Leptosphaeria maculans*. This gene sequence was analyzed further because, although *L. maculans* is not a cereal pathogen, it causes blackleg disease of canola which is a common rotation crop used in cereal farming systems [31], and *L. maculans* is related to other cereal pathogens in the *Pleosporales* family [32], [33]. Again, phylogenetic analysis supported the relatedness of these fungal genes to bacterial sequences (Figure S2). However, any potential acquisition from bacteria is clearly extremely ancient as the sequence seems to have undergone considerable vertical diversification within the fungi following acquisition from bacteria. This hypothesis is not only supported by the phylogenetic analysis but also computational predictions, suggesting that the position and number of introns in *FPSE_11233*-like sequences are not conserved between fungal species (data not shown). The placement of the *L. maculans* sequence outside of the Dothideomycete clade in the phylogenetic analysis (Figure S2) may also indicate a more complex mode of inheritance within the fungi.

A number of other enzyme-encoding genes of putative bacterial origin were also identified in the *F. pseudograminearum* genome, including genes encoding a hydrolase of unknown specificity (FPSE_07775) and a NAD+ dependent dehydratase/epimerase (FPSE_11221). Two putative amidohydrolase encoding genes (FPSE_00725 and FPSE_05717) that have clear bacterial homologues were also identified in this analysis. One of the amidohydrolase genes, FPSE_05717 hereafter termed *FpAH1*, also appeared to have a clear homologue in *P. nodorum* but not in any other fungus, suggesting a potential role for this gene in cereal pathogenesis and an unusual evolutionary history. The genomic organization, sequence diversity and virulence function of *FpAH1* will be described in more detail later in the manuscript.

Many *F. pseudograminearum* genes also had homologues in other fungal cereal pathogens but no clear bacterial matches, suggesting that these genes are either laterally inherited or rapidly diversifying and therefore have been selectively retained only in a limited number of pathogenic fungi. Examples of these (Table 3) include three genes (*FPSE_06956*, *FPSE_10646* and *FPSE_02381*) encoding small secreted proteins (candidate effector molecules) that were present in *F. pseudograminearum* and *F. graminearum* as well as other cereal pathogens. FPSE_06956 had orthologous matches in the *Magnaporthe* and *Fusarium* lineages but no other matches in the BLASTmatrix or GenBank. FPSE_10646 is a member of the killer protein 4 family (PFAM09044) of toxins that were shown to be extensively laterally shared between multiple fungal lineages including non-pathogens [34]. FPSE_02381 is a member of a two-gene family encoding small secreted cysteine rich proteins in *F. pseudograminearum* and *F. graminearum* and has strong, albeit non-reciprocal BLASTp matches in *M. oryzae*. The other member of this family in *F. pseudograminearum*, FPSE_05488, had reciprocal best BLASTp hits only in cereal pathogens, with the exception of a single match in the canola pathogen *Colletotrichum higginsianum* in the BLASTmatrix analysis (Dataset S1). Three other gene products shown in Table 3 (FPSE_05718, FPSE_05719 and FPSE_05720) were encoded in a gene cluster in the *F. pseudograminearum* genome. Interestingly, *FpAH1*, which is predicted to be of bacterial origin (see above and Table 2), also seems to be part of this cluster. The function of *FpAH1* will be described in more detail later in the manuscript.

Also shown in Table 3 is a gene encoding a putative dienelactone hydrolase (FPSE_08136), hereafter termed *FpDLH1*, with very strong orthologues in *F. verticillioides* and *C. graminicola* with 98% and 88% identities, respectively at the amino acid level. The next best BLASTp match (69% identical) of FpDLH1 in GenBank, which is not a reciprocal best hit, is to FGSG_00089 from *F. graminearum*. The absence of strong reciprocal matches in all other fungi suggests that this gene may have been either shared between, or independently acquired from another donor species, by these *Fusarium* and *Colletotrichum* species through horizontal transfer mechanisms. The genomic organization and function of *FpDLH1* will be described in more detail later in the manuscript.

### *FpAH1*, a Horizontally Acquired Amidohydrolase Gene

**Figure 2:**
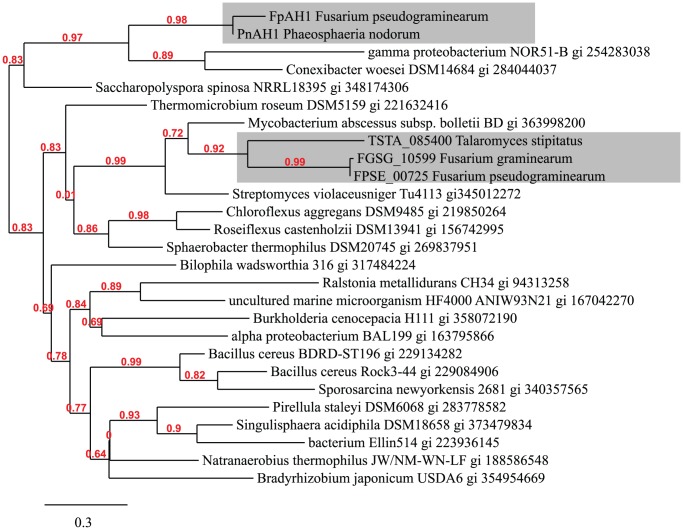
Phylogram of fungal amidohydrolases found in *F. pseudograminearum* that appear to be of bacterial origin and a number of bacterial amidohydrolases. Fungal sequences are highlighted in grey boxes. Numbers on branches indicate approximate likelihood ratio test branch support values.

*FpAH1* encodes a putative amidohydrolase (Pfam domain PF07969). The best BLASTp match (e-value 2e^−141^, 88% identical, 94% similar at the amino acid level) of FpAH1 was to a predicted protein from the wheat glume blotch pathogen *P. nodorum* (encoded by *SNOG_04819* hereafter termed *PnAH1*) [35]. The lack of *FpAH1* homologues in other fusaria and presence in *P. nodorum* was confirmed via hybridization of a *FpAH1* probe to genomic DNA of various *Fusarium* and a *P. nodorum* isolates (Figure S3). The overwhelming majority of subsequent BLASTp matches of FpAH1 in GenBank were to predicted proteins from bacteria (Table 4). The next fungal match after *P. nodorum* was to the *F. graminearum* protein FGSG_10599 (25% identical 2e^−25^) but this was much weaker than the bacterial matches (Table 4). FGSG_10599 also had another much stronger, indeed nearly identical (97%) protein (FPSE_00725) encoded in the *F. pseudograminearum* genome. The FPSE_00725/FGSG_10599 orthologs also had next best matches in bacteria. Phylogenetic analysis of these two *F. pseudograminearum* amidohydrolases strongly supports the hypothesis that they are of bacterial origin (Figure 2).

**Table 4:** Summary of BLASTp analysis of FpAH1 versus the non-redundant protein database (nr) at NCBI.

Hit number†	Accession#	Species	% aa identity (similarity)	coverage of query (%)	e-value
1	EAT87210 (SNOG_04819	*P. nodorum*	88 (94)	100	∼0
2	EED35590	Gamma proteobacterium NOR51-B	38 (58)	96	1e^−136^
3	EIG62154	*Bradyrhizobium* sp. WSM1253	37 (55)	98	1e^−131^
4	ADB51002	*Conexibacter woesei* DSM 14684	38 (57)	96	9e^−126^
5	ABZ07000	uncultured marine microorganism HF4000_ANIW93N21	26 (46)	98	9e^−47^
161	XP_390775 (FGSG_10599)	Second fungal hit (*F. graminearum*)	25 (42)	96	1e^−23^
>250	EED21309	Third fungal hit (*Talaromyces stipitatus*)	24 (40)	91	2e^−20^

† BLASTp performed 18-May-2012.

The *F. graminearum* genome contains six entries in this class of amidohydrolases (PF07969), all of which contain predicted orthologous matches with other genes in the *F. pseudograminearum* genome (Table S2). In other fusaria, clear orthologous relationships exist between the remaining five amidohydrolases with Pfam domain PF07969 found in *F. pseudograminearum*, although both *F. oxysporum* and *F. verticillioides* contain additional members in this class of amidohydrolases (Table S2). In *P. nodorum*, only four proteins fall into this class of amidohydrolases. Thus *FPSE_00725* and *FpAH1* encode two amidohydrolases with extremely restricted distribution in fungi and are most likely of bacterial origin. BLASTp analysis of the other *F. pseudograminearum* amidohydrolases identified a much wider distribution in fungi with FPSE_00474, FPSE_02365, FPSE_03227 and FPSE_11444 having more than fungal 30 hits of greater strength than the best bacterial hit (Table S2). FPSE_05738 was less widely distributed with seven hits of greater score than the best bacterial hit (Table S2) and may also be a candidate for acquisition from bacteria.

The predicted FpAH1 protein of 570 amino acid residues was encoded by an uninterrupted open reading frame of 1710 bp that was confirmed by RNAseq analysis of cDNA (data not shown). The *P. nodorum* genome annotation for *PnAH1* contained a single intron, but it is likely that this prediction was not correct as the *PnAH1* genomic region has a single uninterrupted open reading frame. In the coding region, *FpAH1* was conserved between *F. pseudograminearum* and *P. nodorum* with 89% identity at the nucleotide level and 174 bp upstream of the predicted start codon and 85 bp downstream of the predicted stop codon could also be readily aligned.

**Figure 3:**
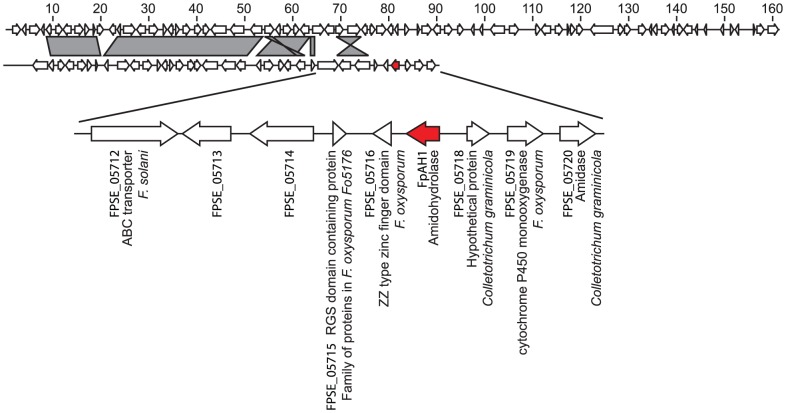
Nucleotide alignment of the end of *F. graminearum* chromosome 1 (top) and the *F. pseudograminearum* contig containing *FpAH1* (bottom). Clear orthologous relationships were evident between regions joined by grey shaded boxes. *FpAH1* is indicated in red. The genes surrounding *FpAH1* in the *F. pseudograminearum* genome that do not have clear orthologous relationships in *F. graminearum* are blown up and the putative function and species of the closest match in GenBank is indicated.

The chromosomal complement of *F. pseudograminearum* has not been characterized and therefore the location of *FpAH1* in the *F. pseudograminearum* genome is unknown. *FpAH1* is present near the end of a ∼90 kb sequence contig, the first 70 kb of which aligns with the end of *F. graminearum* chromosome 1, as shown in Figure 3. Interestingly, the genes surrounding *FpAH1* did not have clear orthologues in the *F. graminearum* genome. On the contig containing *FpAH1*, 28 genes (*FPSE_05686* through to *FPSE_05714*, excluding *FPSE_05712*) had clear orthologues in *F. graminearum*. However, *FPSE_ 5715* to *FPSE_05720* did not. Furthermore, three of the gene products (FPSE_05718, FPSE_05719 and FPSE_05720) were also identified in the BLASTmatrix analysis to be cereal pathogen-specific, suggesting that parallel selection or coordinated acquisition may have affected this region (Table 3). The putative function and position of the genes in this region in *F. pseudograminearum* are shown in Figure 3. Of the remaining 40 genes on the end of chromosome 1 in *F. graminearum*, only eight encoded proteins with reciprocal best BLAST hits in *F. pseudograminearum*, and these were distributed across six different contigs in the *F. pseudograminearum* assembly (data not shown). In contrast, *PnAH1* is ∼100 kb from the end of supercontig seven of the *P. nodorum* genome and appears to be surrounded by genes encoding proteins conserved in other fungi. In both *P. nodorum* and *F. pseudograminearum*, the GC content in the region of the *PnAH1* and *FpAH1* was similar to that of other gene rich regions of the respective genomes and some of the surrounding genes in the regions had introns. In summary, the genomic region containing *FpAH1* has no equivalent sequence at the syntenic location in the *F. graminearum* genome nor the region containing *PnAH1* in the *P. nodorum* genome.

### *AH1* Sequence Phylogenies Indicate Ancient and Independent Horizontal Transfers into Fungal Pathogens

The lack of close orthologues of *FpAH1* and *PnAH1* in other fungi and the presence of similar genes in bacteria (Table 4) based on BLASTp searches suggested an origin for these fungal genes via horizontal acquisition from bacterial species. fusaria belong to the order/class Hypocreales/Sordariomycetes while *Phaeosphaeria* is in the distantly related Pleosporeales/Dothidiomycetes. Acquisition of the gene may have occurred independently in both species or alternatively was horizontally transferred between the fungal species. To differentiate these possibilities, the sequence diversity of each gene was assessed in several globally sourced *Fusarium* and *Phaeosphaeria* isolates (Table S3). Isolates identified as *F. pseudograminearum* by multi-locus DNA sequencing [22] or by sequencing the *elongation factor 1 alpha* (*EF1α*) gene, were the only *Fusarium sp.* out of six tested that produced positive amplicons. PCR analyses of the distribution of *FpAH1* in fusaria were confirmed for a limited number of isolates by hybridization analysis (Figure S3). *PnAH1* sequences were PCR amplifiable from all *P. nodorum* isolates tested and also from two of four sister species, *P. avenaria* f. sp. *tritici* (*Pat*) *1* and *Pat3*, suggesting *PnAH1* was present in a common ancestor in this lineage.

**Figure 4:**
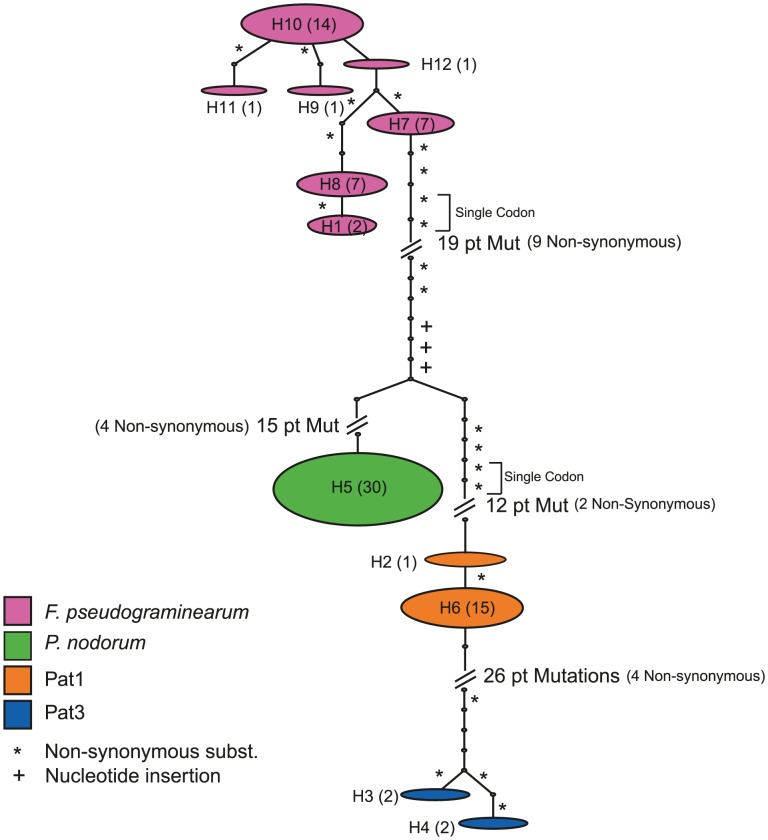
Statistical parsimony haplotype network for *FpAH1*/*PnAH1* in *Fusarium pseudograminearum* and *Phaeosphaeria* spp. Haplotype bubbles are proportional to sample size shown in parentheses. Dots on line segments indicate single point mutations.

In order to test the hypothesis of horizontal transfer between *F. pseudograminearum* and *P. nodorum*, a ∼500 bp region of the *AH1* gene was sequenced in isolate collections. A haplotype network showing the sequence relationships across both genera is shown in Figure 4. In *F. pseudograminearum*, seven haplotypes observed did not correspond to the geographic origin of isolates, consistent with global gene flow within the species as previously described [22]. Diversity in the *Phaeosphaeria* spp. sequences was more limited with only one haplotype observed in a global sample of *P. nodorum* and two haplotypes detected within each of the two *Phaeosphaeria* sister species containing *PnAH1* orthologues. There were no shared sequence haplotypes between *Phaeosphaeria* spp. and *F. pseudograminearum*. The presence of *AH1* orthologues in up to four related *Phaeosphaeria* spp. suggests that the acquisition of this gene occurred before the divergence of these species. Divergence of *PnAH1* between the *Phaeosphaeria* spp. was comparable to divergence observed at neutral sequence loci (M.C. McDonald, unpublished data). Furthermore, the haplotypes observed in the *Phaeosphaeria* species complex and *F. pseudograminearum* were distinct, suggesting independent acquisitions of *AH1*-like sequences by each lineage.

### *FpAH1* Contributes to Virulence on Wheat and Barley

**Figure 5:**
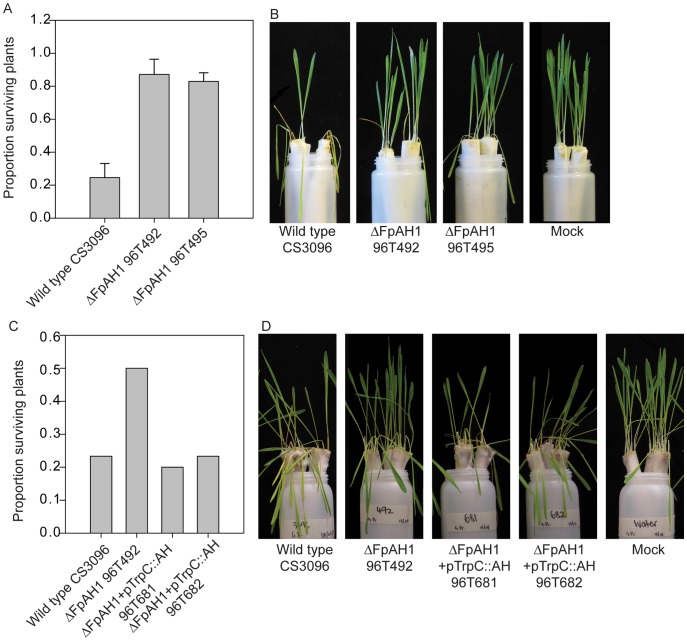
Virulence assay of the *Fusarium pseudograminearum* amidohydrolase 1 mutants (ΔFpAH1) compared to the parental strain towards barley cultivar Golden Promise at 21 days post inoculation (dpi) (A–B). (A) Survival of plants in the assay 21 dpi. N = 3 with each replicate consisting of 13–15 plants divided between two paper towel rolls and maintained in separate vessels. The difference between wild type and each mutant was highly statistically significant (P-value <0.01). (B) Representative rolls of plants from the assay. (C–D). Complementation of ΔFpAH1 (strains 96T681 and 96T682) restores virulence towards barley cultivar Golden Promise. (C) Proportion of plants surviving at 13 dpi. The total number of plants in each case was 30. (D) Photograph of assays at 13 dpi.

The likely independent acquisition and retention of *AH1* orthologues by *F. pseudograminearum* and *P. nodorum* suggests that this gene may encode a protein that is necessary for virulence, at least in some hosts. To test this hypothesis, a functional analysis of the *FpAH1* and *PnAH1* genes was undertaken. *FpAH1* was expressed in both infected barley and wheat leaves and roots (Figure S4). The role of the *FpAH1* amidohydrolase in FCR of barley was also assessed by generating gene deletion mutants in *F. pseudograminearum* by replacing 635 nucleotides of the *FpAH1* locus with a geneticin resistance gene cassette (Figure S5A). There were no obvious defects in appearance or sporulation of the mutants. A culture time-course was used to compare the growth rates of one mutant to that of the parental strain and these were indistinguishable (Figure S5C). As shown in Figure 5, two independently-derived knock-out mutants consistently showed reduced virulence towards barley (cv. Golden Promise) seedlings across multiple independent experiments using a previously established FCR inoculation assay [36]. A second barley cultivar (cv Gairdner) also showed similar reduced virulence (data not shown). FCR disease severity caused by the mutant strains was significantly (*P*-value<0.01) reduced compared to those caused by the wild type strain. Genetic complementation of an *FpAH1* mutant with a cassette containing *FpAH1* under the control of the *Aspergillus nidulans TrpC* promoter restored virulence towards barley (Figure 5C and 5D), providing further evidence that *FpAH1* is required for virulence against barley.

**Figure 6:**
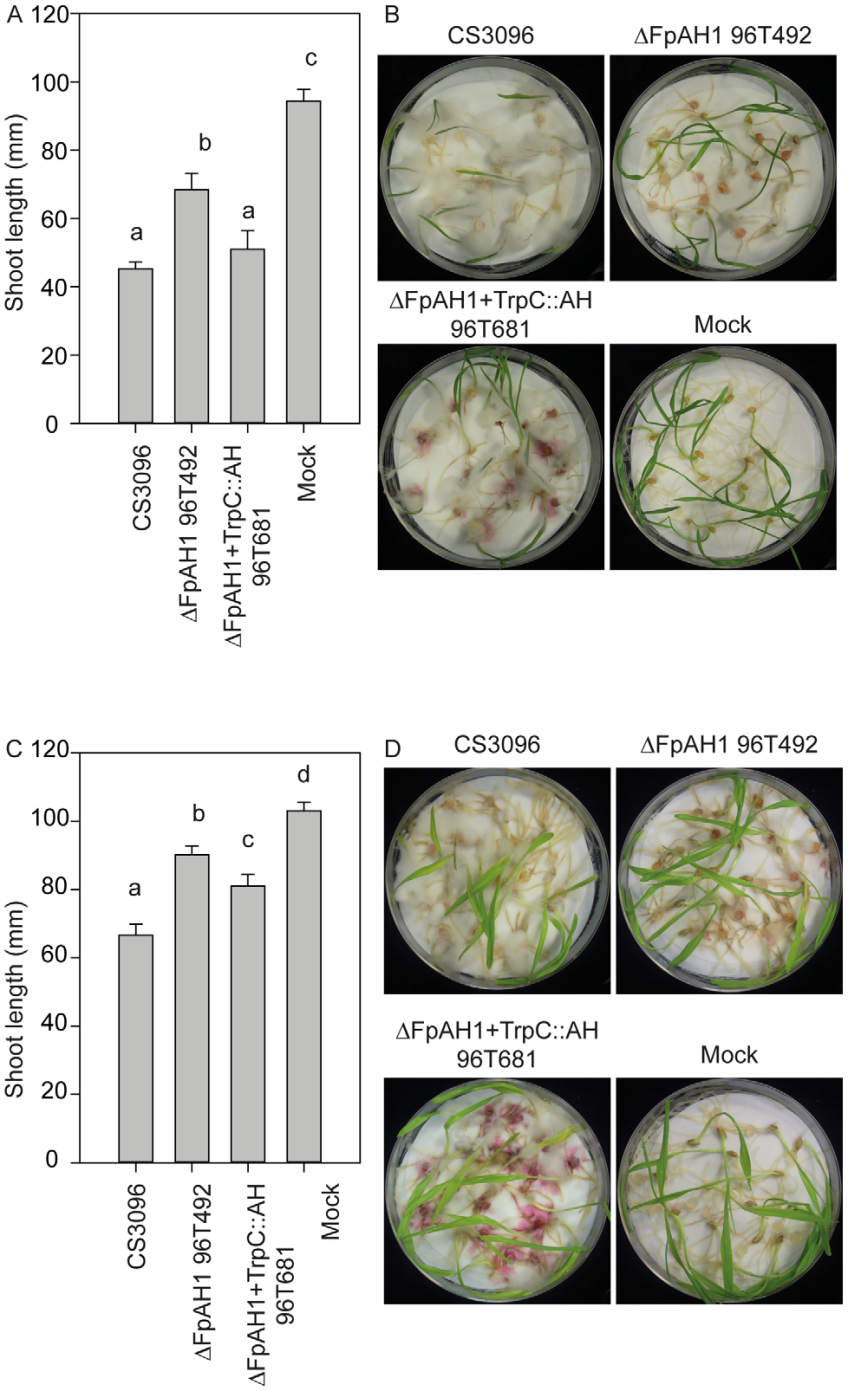
Fusarium root-rot virulence assay of the *Fusarium pseudograminearum* amidohydrolase 1 mutant (ΔFpAH1) towards wheat cultivar Kennedy (A and B) and barley cultivar Golden Promise (C and D). N = 16 individual plants. CS3096 is the parental strain for the FpAH1 mutant (96T492), which was complemented with *FpAH1* driven by the *TrpC* promoter (96T681). Error bars represent the standard error of the mean. Different letters on A and C indicate statistically significant differences (*P*<0.05) in pair-wise t-tests. B and D are the plants used to score the shoot length shown in A and C.

*F. pseudograminearum* has a wide host range within cereals with no evidence for race specialization [37], [38] for FCR disease on wheat. Unlike the experiments conducted on barley, highly replicated FCR assays on wheat using the *FpAH1* knock-out mutants failed to reveal reduction of virulence on two unrelated varieties of wheat (Figure S6). However, reduced virulence was detectable when inoculated directly on roots toward both wheat and barley and again complementation restored virulence (Figure 6). *P. nodorum* is not pathogenic on barley and therefore was not tested on this host but mutant strains of this pathogen with deletions of the *PnAH1* gene were also generated (Figure S5D) and tested on wheat plants in replicated leaf infection assays. No significant differences in virulence were observed between mutant and wild type strains on wheat (Figure S7). These experiments indicate that *FpAH1* is required for full virulence on both wheat and barley but the importance of *PnAH1* in pathogenesis remains unknown.

### Novel Genomic Context and Virulence Function on Cereals for the *FpDLH1* Gene

**Figure 7:**
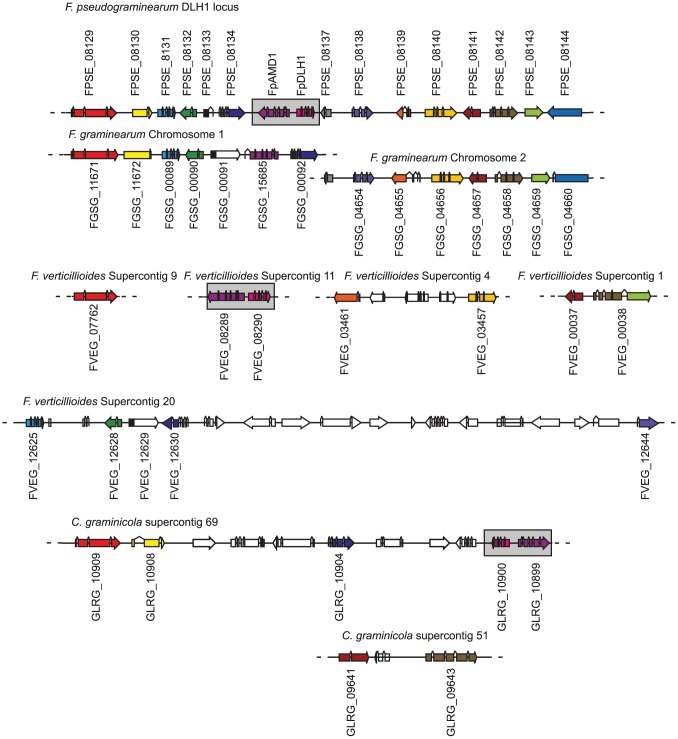
The *FpDLH1*-*FpAMD1* locus in *Fusarium pseudograminearum* and the arrangement of the genes in this region in *F. graminearum*, *F. verticillioides* and *Colletotrichum graminicola*. The *DLH1*-*AMD1* locus is boxed in grey in each of the species. Genes of the same color in different species are reciprocal best blast hits and likely to be orthologous. Sequence contigs for each species are truncated to the relevant regions only.

The *F. pseudograminearum* locus *FpDLH1* encoding a dienelactone hydrolase was identified in the BLASTmatrix analysis as a cereal-pathogen associated gene with homologues detected only in *F. verticillioides* and *C. graminicola*. Strikingly, the gene (*FPSE_08135* hereafter termed *FpAMD1*) immediately adjacent to *FpDLH1* in the *F. pseudograminearum* genome encodes an amidase that is also present in the same divergently transcribed arrangement in the genomes of *F. verticillioides* and *C. graminicola* (Figure 7), indicating this is likely to be a two-gene cluster. The amidase family is much larger in ascomycetes than the dienelactone hydrolase family, making one-to-one orthologous relationships between species difficult to detect. However, for both *FpAMD1* and *FpDLH1*, strong homology is detected between all three species at the nucleotide level (>80%) across the coding sequences of these genes. Furthermore phylogenetic analysis also supports the grouping of both of these genes in the three species in clades incongruent with expected evolutionary relationships (Figure S8). Thus, their genomic arrangement, discontinuous distribution in the fungi and strong homology to each other all suggest these genes represent a two-gene cluster that may have a common origin.

The *FpDLH1* gene has the same intron-exon structure as another dienelactone hydrolase that is present in both *F. pseudograminearum* and *F. graminearum* (*FPSE_08131* and *FGSG_00089*, Figure 7). This intron arrangement is not a generic feature of dienelactone hydrolase encoding genes in *F. pseudograminearum*, indicating a possible gene duplication event in the *Fusarium* lineage. Furthermore, FPSE_08131 had reciprocal best BLAST hits in all fusaria included in the analysis, with the exception of *F. solani*, as well as in two *Magnaporthe* spp., and the intron-exon structure was maintained in all species. The synteny in this region is also well conserved in the *F. pseudograminearum*-*F. graminearum* comparison (albeit split into two regions that align near the ends of different chromosomes in *F. graminearum*) but outside of this comparison the synteny weakens with the orthologues of the genes flanking this two-gene cluster in *F. pseudograminearum* appearing on multiple different contigs in *F. verticillioides* (Figure 7). In the *F. verticillioides*-*F. pseudograminearum* comparison, the conservation of FpDLH1/FvDLH1 (98% identical at the amino acid level) is much greater than the more widespread dienelactone hydrolase (FPSE_08131 and its *F. verticillioides* orthologue FVEG_12625) at 75% identity, suggestive of either an intra-fusaria transfer or strong conservation and selection. In *C. graminicola* the *DLH1*-*AMD1* gene cluster is located on chromosome 2 in a region where only two of 11 genes flanking the cluster have clear orthologous relationships between *C. graminicola* and the closely related *C. higginsianum* (data not shown). However, three of the nine genes on one flank of the *C. graminicola DLH1*-*AMD1* gene cluster have orthologues present in the flank adjacent to *FpDLH1*-*FpAMD1* (Figure 7). These genomic associations suggest an ancient relationship in these regions.

**Figure 8:**
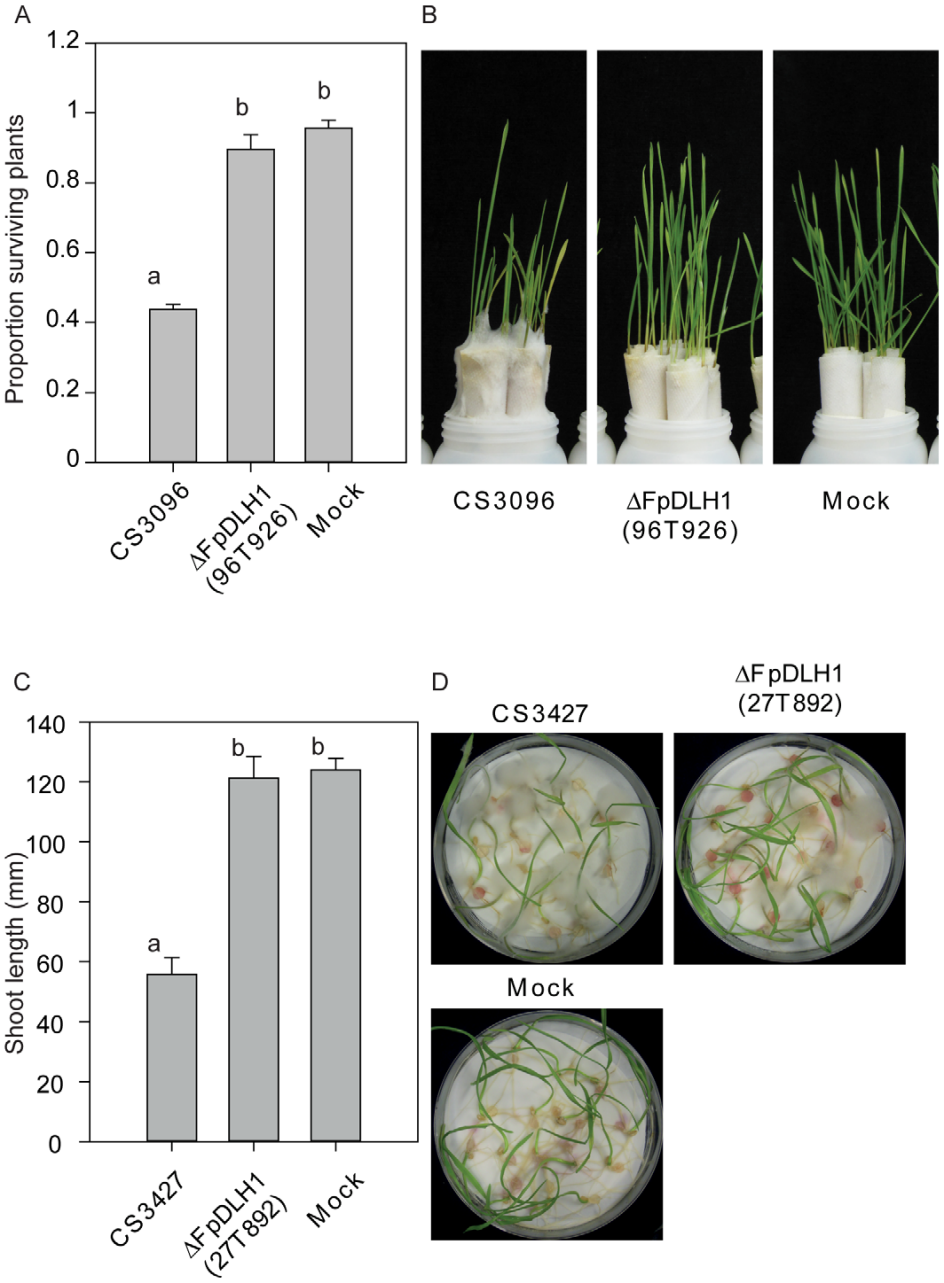
Virulence assay of the *Fusarium pseudograminearum dienelactone hydrolase 1* mutant (ΔFpDLH1) mutant towards wheat cultivar Kennedy in crown rot (A and B) and root rot (C and D) assays. For A and B N = 3 with each biological replicate consisting of 4 paper towel rolls consisting of 7–8 plants per roll. For C and D N = 15–16 plants. Error bars represent the standard error of the mean. Letters indicate statistically significant differences at *P*<0.05. CS3096 and CS3427 are the parental strains for the FpDLH1 mutants, 96T926 and 27T892 respectively. Mock treatments are inoculations performed with agar plugs that have not been colonized by *Fusarium*.

Both *FpDLH1* and *FpAMD1* are expressed during infection of root by *F. pseudograminearum* and leaf tissue with higher expression in wheat than in barley (Figure S4). The role of *FpDLH1* in fungal pathogenesis was assessed by creating knockout strains of two different *F. pseudograminearum* strains (CS3096 and CS3427), where the *FpDLH1* gene was replaced by the geneticin resistance cassette (Figure S9). No obvious differences in sporulation were observed in the mutants nor were there differences in growth rate compared to their respective parents (Figure S9C). However, *FpDLH1* mutants in both strain backgrounds showed significantly reduced virulence towards both wheat and barley in both root-rot and FCR assays (Figure 8 and Figure S10), suggesting that FpDLH1 contributes to fungal virulence against cereal plants.

## Discussion

The new DNA sequencing technologies are well suited to characterizing low copy, gene-rich regions of fungal genomes that are relatively small in size. Using Illumina technology it was possible to assemble *de novo* an almost complete sequence of the *F. pseudograminearum* genome. A large proportion (∼94%) of the *F. pseudograminearum* genome showed high similarity to *F. graminearum* and a great deal of co-linearity was observed. Alignment of the *F. pseudograminearum* genome sequence to the chromosomes of *F. graminearum* revealed that regions of poor sequence match were concentrated in specific regions, such as the ends of chromosomes and what are thought to be regions of ancient chromosome fusion in this *Fusarium* lineage and probable regions of genome innovation [2], [28]. Although more strains will need to be sequenced to confirm the species-specificity of these regions of the *F. pseudograminearum* genome, it is possible that the genes contained in these regions may be responsible for various phenotypes that distinguish *F. pseudograminearum* from *F. graminearum*, such as its propensity to cause FCR rather than FHB, its broad geographical adaptation in arid cereal production areas [39].

We hypothesized that genes in the *F. pseudograminearum* genome that were either uniquely or predominantly present in other cereal fungal pathogens may have a specialized function related to cereal pathogenesis and niche specialization. To identify these genes, we undertook a BLASTmatrix analysis and found that many genes present in the *F. pseudograminearum* genome were also present exclusively in cereal pathogens. 214 of these genes appeared to be conserved in the three cereal-infecting fusaria but had no equivalent matches in the genomes of three fusaria that infect dicotyledonous plants. These genes may have undergone specialized selection in these cereal pathogen *Fusarium* lineages but been diversified or lost in other fusaria. Several *F. pseudograminearum* genes also appeared to have equivalents in cereal pathogens outside the fusaria, and these were present mostly in other necrotrophic or hemibiotrophic Ascomycete fungi. Two of these genes, encoding an amidohydrolase (FpAH1) and a dienelactone hydrolase (FpDLH1) were selected for functional analysis in *F. pseudograminearum*. In both cases, we demonstrated roles in virulence on cereal hosts. This suggests that the comparative genomic analysis that we undertook is a powerful approach for the identification of genes with specialized pathogenesis on related hosts. Our analyses also identified many other candidate *F. pseudograminearum* genes showing similar distributions and a systematic analysis of their functions in fungal virulence is now warranted.

Our analysis identified genes in the genome of *F. pseudograminearum* with matches in bacterial genomes, and a number of genes were restricted to other fungal pathogens of cereals. These findings are consistent with possible acquisition of these genes by horizontal transfer. These observations also suggest considerable genome plasticity in *F. pseudograminearum* and provide a number of candidates for further study of potential horizontal acquisition. Compelling evidence for acquisition of a gene of bacterial origin and retention in cereal-infecting fusaria is illustrated by *FPSE_07765* encoding a putative aminotransferase. Most matches to this aminotransferase were from bacteria, with the closest match showing a remarkable 75% amino acid identity to a predicted protein in the genome of *Microbacterium testaceum*, a common bacterial endophyte of cereals. Horizontal transfer from co-habiting endophytic bacteria into the *Fusarium* lineage with selective retention in cereal pathogens is a simple explanation for these strong, but restricted gene homologies and organismal relationships. Another significant bacterial match was the putative cell wall hydrolase encoded by *FPSE_11233*, with equivalent proteins present in many cereal pathogens. In this case, phylogenetic analysis indicated that all identified proteins from diverse fungal species clustered into a single clade. The biased occurrence of this gene in plant pathogens is likely due to selective retention after an ancient acquisition event during fungal evolution.

*FpAH1* represents a striking example of likely horizontal movement of a gene from a bacterium into the *F. pseudograminearum* genome. The only closely related gene to *FpAH1* in the fungi examined was *PnAH1* found in the genome sequence of *P. nodorum*, a pathogen of wheat. Sequencing of several globally distributed isolates of *F. pseudograminearum* revealed several distinct haplotypes for this gene. This suggests that acquisition of *FpAH1* was not recent or that there has been significant selection driving the creation of new alleles. There was no evidence of horizontal transfer directly between *F. pseudograminearum* and a *Phaeosphaeria* spp. The limited divergence of *PnAH1* between the *Phaeosphaeria* spp. orthologues, suggests that a common ancestor of these *Phaeosphaeria* species acquired the gene. The presence of the gene in at least two lineages of cereal pathogens suggests that the gene may play an important role in wheat pathogenesis.

Both *FpAH1* and *PnAH1* were clearly dispensable for growth and their retention in two otherwise unrelated fungal pathogens suggests a specialized function for these genes in fungal virulence. Indeed, a virulence function for FpAH1 against two different cereal hosts was confirmed. However, *PnAH1* knockout strains did not show altered virulence against wheat. Pathogenesis in any one species is the sum of many different components and the relative contribution of these genes to pathogenesis in the different species may be substantially different. The genomic context of the genes in these two fungal pathogens was also very different. *PnAH1* is located in a region of the *P. nodorum* genome adjacent to several conserved genes. In contrast, *FpAH1* occurs at the end of a long contig in a cluster of genes found in other cereal pathogens, but not in *F. graminearum*, and it could be that *FpAH1* is functioning in *F. pseudograminearum* as part of this group of genes. It appears that most of the genes on the end of chromosome 1 in *F. graminearum* are absent from *F. pseudograminearum*. This observation provides further support to the notion that chromosome ends or ancient chromosome fusion sites are regions of genome innovation in the *Fusarium* lineage [28] and regions like this may have played a role in niche separation between *F. pseudograminearum* and *F. graminearum*. The role and reason for retention of *PnAH1* in *P. nodorum* however, remains unknown.

The two-gene cluster represented by *FpDLH1*-*FpAMD1* is likely to be of fungal origin in the genomes of *F. pseudograminearum*, *F. verticillioides* and *C. graminicola*. A comparison of the genomic context of the *DLH1-AMD1* genes in *F. pseudograminearum* and *C. graminicola* also supports a common origin with some related genes being present in flanking regions of this cluster in both pathogens. The conservation (∼80%) of the nucleotide sequence across the coding regions of both genes between *C. graminicola* and the two *Fusarium* spp. suggests that any possible genetic exchange event between these lineages is ancient and has thereby allowed accumulation of considerable sequence divergence. The close physical proximity in the *F. pseudograminearum* genome of *FpDLH1* to another dienelactone hydrolase encoding gene, *FPSE_08131*, with identical intron-exon structure and with orthologues in other fusaria, suggests *FpDLH1* may have arisen vertically by gene duplication and subsequent diversification within the *Fusarium* lineage. The FpDLH1/FvDLH1 and FpAMD1/FvAMD1 orthologues of *F. pseudograminearum* and *F. verticillioides* show remarkable identity (98%) at the amino acid level but very different genomic context. However, currently, it is not possible to resolve whether an inter-species transfer had occurred between the *Fusarium* lineages, or alternatively whether strong *DLH1* and *AMD1* gene conservation with regional genomic rearrangements had occurred in these two cereal infecting fusaria. Further diversity surveys will be required to resolve the origin of *FpDLH1*- and *FpAMD1*- related genes in these cereal pathogens.

Both FpAH1 and FpDLH1 are thought to be catabolic enzymes based on conserved domain matches. Amidohydrolases are a diverse superfamily of enzymes that catalyze the hydrolysis of C-N bonds in small molecules. This family includes enzymes functioning in central metabolism (eg urease), enzymes that degrade xenobiotics (eg atrazine) as well as those that are known to catalyze reactions other than C-N cleavage, including P-O cleavage and also isomerisation [40]. Likewise, the dienelactone hydrolase family of enzymes appears to be large, with members involved in the degradation of chloroaromatic compounds [41], [42] by bacteria and activation of prodrugs containing lactone-like side chains in humans [43]. The specific biochemical roles of FpAH1 and FpDLH1 during this particular host-microbe interaction remain elusive. However, the predicted catabolic activity of these enzymes suggests they could be either targeting specific plant defense compound(s) or involved in production of fungal toxin(s). Although the defensive compounds important in the response to *F. pseudograminearum* are currently unknown in barley and wheat, candidates may include hordatine, hordenine and gramine in barley and the benzoxazolinones in wheat [44]–[47]. All these compounds contain C-N bonds and are known to have antifungal properties [44]–[47]. However, fungal growth inhibition assays conducted in liquid culture medium with synthetic hordatine and gramine showed that while *F. pseudograminearum* is moderately sensitive to both compounds, *FpAH1* knockout strains were equally as sensitive as the wild type (data not shown). Likewise, the *FpDLH1* mutants were as sensitive as the wild-type to the benzoxazolinones, BOA (2-3H-Benzoxazolinone) and MBOA (6-methoxy-2(3)-benzoxazolinone). However, in *F. verticillioides* BOA detoxification is a two-step process with only one of the two responsible genes having been cloned [48]. A more detailed understanding of the barley and wheat metabolites involved in defense against *F. pseudograminearum* would be an important starting point to identify molecular mechanism(s) of FpAH1- and FpDLH1- mediated virulence.

Genomics is allowing increased discovery of examples of likely horizontal gene transfer events [6], [7]. Our work provides additional evidence for this hypothesis although detection of horizontal gene transfers, particularly of ancient events, requires robust phylogenetic analyses [3], [7], [49]. The extent of the horizontal gene transfer phenomenon has not yet been fully ascertained amongst the sequenced fungal genomes but there will presumably be many more examples. A robust methodology needs to be developed to enable identification of horizontally acquired genes on a scale of kingdoms and beyond. The BLASTmatrix analysis presented here is one possible method for identifying these genes, although it is limited in its ability to cope with post-acquisition family expansion. It is also important to note that the analysis reported here was centered on *F. pseudograminearum* and limited by the species included in the comparative analyses. A broader systematic analysis for each fungal pathogen genome is warranted to obtain a more complete perspective of potential gene sharing and its relation to host range and virulence.

In summary, in this paper we report the first genome sequence for *F. pseudograminearum* and demonstrate that a broad comparative genomic analysis can identify genes that show a biased distribution in fungal pathogens of plants with putative roles in virulence processes or niche specialisation. Functional analysis in *F. pseudograminearum* of two such genes demonstrated novel virulence functions on cereals.

## Materials and Methods

### Fungal Isolates and Culture

The *F. pseudograminearum* isolate (CS3096) chosen for genome sequencing was originally isolated from a wheat crown collected in 2001 near Moree in Northern New South Wales, Australia [26]. Isolates for phylogenetic analysis of the *FpAH1* gene were selected from a collection housed at CSIRO Plant Industry, Brisbane, Australia, consisting of isolates from Australia, New Zealand, Canada, United States of America and Turkey (Table S3). *Phaeosphaeria* isolates were selected from a collection housed at ETH Zurich and the Australian isolate SN15.

### Sequencing and Assembly

Two lanes of single-end 75 bp and one lane of 100 bp paired-end sequencing was performed on an Illumina GAII_x_ genome sequencer by the Australian Genome Research Facility, Melbourne Australia. Reads were imported into CLCBio Genomics Workbench 3 and quality trimmed (default parameters) prior to assembly. A total of 60 million paired reads and 33 million single end reads were used in *de novo* assembly plug in (version 3.03), again using default parameters with a minimum contig size of 500 bp. Low coverage (<40×) contigs (compared to the genome average of 179×) were excluded from further analyses. BLASTn comparison to the *F. graminearum* mitochondrial sequence and coverage information (3,500–6,000×) was used to separate nuclear and mitochondrial sequence. 29 contigs with total length 107 Kbp were identified in this process and excluded from the genome annotation but are included in the *F. pseudograminearum* genome submission to GenBank. This Whole Genome Shotgun project has been deposited at DDBJ/EMBL/GenBank under the accession AFNW00000000. The version described in this paper is the first version, AFNW01000000.

### Comparison to the *F. graminearum* Genome

Repeat masking was performed using RepeatMasker version open-3.2.8 [50], run in sensitive mode with cross_match version 0.990329 as the search engine and RepBase version 20090604 [27]. The species descriptor “fungi” was used.

Whole genome alignments were performed with the NUCmer and PROmer algorithm in the MUMmer package [51]. The minimum cluster length was set to 100 bp. The total length of the genome alignment between *F. graminearum* and *F. pseudograminearum* was calculated by summing the length of all non-redundant alignments extracted by using the show-coords program (part of the MUMmer package).

### Gene Prediction

Protein coding genes were *ab initio* predicted in the *F. pseudograminearum* genome using FGENESH [52] based on the *F. graminearum* gene models as part of the MolQuest2 package from Softberry, AUGUSTUS [53] and GeneMark-ES version 2 [54]. AUGUSTUS was run with a training set of *F. graminearum* genes that had 100% nucleotide alignment and identity to the *F. pseudograminearum* genome. When the three programs disagreed in the splicing for the same gene, the following strategy was used to prioritize the predictions. When two of the three programs agreed, this prediction was taken. When all three programs differed for what appeared to be the same gene, priority was given in the order FGENESH, GeneMark and AUGUSTUS. Predicted transcripts encoding <20 amino acids were removed manually from the main prediction set. In total 17,503 unique transcripts were predicted, which after removing 4833 alternative predictions for the same gene left a putative set of 12448 unigenes.

### BLASTmatrix Pipelines to Identify Genes Specific to *F. pseudograminearum* or Discontinuously Distributed in Fungi

The proteins coded by 27 different fungal genomes were downloaded from the Broad Institute (www.broadinstitute.org), Joint Genome Institute [55] or NCBI for the *F. oxysporum* strain 5176 genome [56]. Reciprocal best BLASTp hits were used to determine the orthologous protein relationships between *F. pseudograminearum* and each of the 27 fungal protein sets. This was performed using a pair-wise all versus all BLASTp analysis was conducted on the National Computational Infrastructure Specialized Facility in Bioinformatics Cluster located at the University of Queensland, Australia. Perl scripts obtained from http://sysbio.harvard.edu/csb/resources/computational/scriptome/unix/Protocols/Sequences.html were used to extract best reciprocal hits and the associated blast score from each species comparison BLASTp outputs. The *F. pseudograminearum*–*F. graminearum* relationship was treated as a special case and used to curate the dataset. In total, 11481 genes had clear one-to-one relationships between the two species, but differences in gene prediction (eg splice sites or predictions split/fused with respect to each other in each of the genomes) resulted in false identification of genes without orthologous matches in this analysis. These were accounted for by performing BLASTn analysis using *F. pseudograminearum* gene sequences containing introns as the query against the *F. graminearum* genomic sequence. Transcripts showing strong matches to the *F. graminearum* genome (match length greater than 50%) were classified as having different annotation between *F. graminearum* and *F. pseudograminearum*. 456 genes were removed from the analysis using this process.

Logical formulae and filtering functions in Microsoft Excel were used to identify potential cereal pathogen specific proteins via the presence of reciprocal best BLAST hits in one or more of the 16 cereal pathogens at a BLAST bit score of greater than 200 and in the complete absence of a reciprocal best BLAST hit in any of the 11 non-cereal pathogens. Manual curation of putative cereal pathogen specific genes was conducted to account for the possibility that genes were predicted in *F. pseudograminearum* that were missed in the annotation of other genomes. This was performed by querying the intergenic sequences of the nearest non-cereal pathogen, *F. oxysporum* f. sp. *lycopersici* in a tBLASTn search using the putative cereal pathogen specific proteins. Through this process, 112 proteins were removed due to potential missed predictions in the *F. oxysporum* f. sp. *lycopersici* genome.

### Phylogenetic Analyses

Phylogenetic analyses were implemented using phylogeny.fr [57]. Briefly, multiple alignments were generated using MUSCLE [58] with default parameters, and curated using Gblocks [59]. Phylogeny was performed using PhyML [60] with the WAG amino acid substitution matrix [61] using an approximate likelihood-ratio test for branch support [62]. Trees were drawn using TreeDyn [63]. Trees were exported to Adobe Illustrator to allow shading of fungal branches.

### Hybridization Analysis

A slot blot membrane was prepared using a Hoefer PR 648 apparatus using Amersham Hybond-XL membrane (GE Healthcare) prewetted with 0.2 M NaOH. Approximately 500 ng of DNA was prepared in 0.2 M NaOH to a final volume of 60 µL and incubated at 37°C for 15 min prior to application to the membrane. Following application of the DNA the membrane was cross-linked using a GS GeneLinker UV cross-linker (BioRad) with 150 mJ of energy. Two identical membranes were prepared and probed with either a *FpAH1* or *rRNA* probe. Probes were generated using the PCR DIG Probe synthesis kit (Roche) according to the manufacturer's instructions using primers FpAHDiversityF and FpAHDiversityR for the *FpAH1* probe and ITS1-F and ITS4 primers for the *rRNA* probe (Table S4). Hybridization and washing was performed with the DIG Easy Hyb solution and DIG wash and block buffer set (Roche). Detection was performed with the DIG Luminescent detection kit (Roche).

### *FpAH1* and *PnAH1* Diversity Analysis

For *Phaeosphaeria* spp. PCR amplification was performed in 20 µL reactions containing 0.05 µM of PnAHDiversityF and FpAHDiversityR (supplied by Microsynth), 1× Dream *Taq* Buffer (Fermentas), 0.4 µM dNTPs (Fermentas) and 0.5 units of Dream *Taq* DNA polymerase (Fermentas). For *F. pseudograminearum* isolates, the forward primer was replaced with FpAHDiversityF and PCR was conducted using Invitrogen *Taq* DNA polymerase as per the manufacturer's instructions. Primer sequences are listed in Table S4. The PCR cycle parameters were: 2 min initial denaturation at 96°C followed by 35 cycles of 96°C for 30 s, 58°C for 45 s and 72°C for 1 min and a final 5 min extension at 72°C. The same cycling conditions were used for *F. pseudograminearum* isolates except the denaturation was performed at 94°C. *Fusarium*-derived PCR products were purified using the QIAgen PCR purification kit and sequencing was carried out by the Australian Genome Research Facility using both the forward and reverse primers. Sequencing reactions for *Phaeosphaeria*-derived products were conducted in 10 µL volume using the BigDye Terminator v3.1 Sequencing Standard Kit (Applied Biosystems, Foster City, CA) with both the forward and the reverse primer. The cycling parameters were 96°C for 2 min followed by 55 or 99 cycles of 96°C for 10 s, 50°C for 5 s and 60°C for 4 min. The products were cleaned with the illustra Sephadex G-50 fine DNA Grade column (GE Healthcare) according to the manufacturer's recommendations and then sequenced with a 3730×/Genetic Analyzer (Applied Biosystems). Alignment of forward and reverse sequences for each isolate was performed in SeqScape software V2.5 (Applied Biosystems). Translation and identification of protein haplotypes was also performed using this software. The program TCS v1.2 was used to visualize the most-parsimonious haplotype network [64].

### Construction of *FpAH1*, *SnAH1* and *FpDLH1* Mutants

The vectors for deletion of the *FpAH1 and FpDLH1* genes were constructed using lambda phage mediated recombination as described previously [65] using primers listed in Table S4. For *FpAH1* the targeting construct consisted of 1858 bp of sequence immediately upstream of the start codon and 1132 bp covering the 3′ end of the gene and downstream sequence. 635 bp were deleted. For *FpDLH1* the targeting construct consisted of 1523 and 1898 bp flanks, deleting 891 bp of the gene, leaving 125 bp of the 5′ coding sequence and 22 bp of the 3′ coding sequence. *F. pseudograminearum* protoplasting and transformation was performed as previously described for *F. graminearum* [66] except the STC (sorbitol, Tris and calcium chloride solution) was made with 0.8 M sorbitol instead of sucrose and the 40% PEG_8000_ solution also contained 0.8 M sorbitol.

Transformants were selected on 50 mg L^−1^ geneticin (Sigma, St. Louis, MO, USA), subcultured onto geneticin and DNA was prepared using the REDextract and Amp kit as per the manufacturer's instructions (Sigma). Transformants were screened for gene deletion using AHKOscr1, AHKOscr2 and gpdAr primers. In total, 42 transformants were screened and six were identified as carrying a deletion of *FpAH1*. Mutant strains were single-spored and then stored as water cultures from ½ PDA plates.

The *PnAH1* deletion construct was made using overlap PCR as previously described [67]. PnAH1KO5'F and PnAH1KO5'R were used to amplify a 1456 bp region upstream of *PnAH1* whilst PnAH1KO3'F and PnAHKO3'R amplified a 1519 bp region downstream. These flanks were fused using overlap PCR with a hygromycin resistance cassette amplified from pAN7.1, resulting in a deletion construct of 5.9 kb. The deletion cassette was transformed into *P. nodorum* isolate SN15 protoplasts as previously described [68] and screened by using primers designed outside the flanking DNA (PnAH1KOscr-F and PnAH1KOscr-R). Copy number of the transformed construct was determined as previously described [69].

### Complementation of *FpAH1* Mutant

The ΔFpAH1 mutant was complemented by co-transformation of ΔFpAH1 mutant 96T492 with pAN7.1 [70] and a construct designed to expresses *FpAH1* under the control of the *Aspergillus nidulans TrpC* promoter. This construct was created by PCR amplification of the coding sequence and 341 bp of terminator of *FpAH1* from genomic DNA of isolate CS3096 with forward and reverse primers (AH-OXf and AH-OXr) containing *Cla*I and *Eco*RI sites respectively and replacement of the hygromycin cassette in pUChph [71] using the same restriction sites. The *TrpC*-*FpAH1* section of the construct was fully sequenced prior to transformation into ΔFpAH1 mutant 492. Selection was with hygromycin at 200 mg L^−1^. To verify that transformants were over-expressing the *FpAH1* gene after successive rounds of sub-culture and single-sporing, they were grown in 6-well tissue culture plates in liquid basal media with 10 mM (NH_4_)_2_HPO_4_ as the nitrogen source [72]. Mycelia were harvested, freeze-dried and RNA extracted using TRIzol (Invitrogen) according to the manufacturer's instructions after four days of growth at 28°C in the dark. Relative *FpAH1* gene expression was measured using quantitative reverse transcriptase PCR as previously described [73]. *β-tubulin* and *FpAH1* (FpAH1f and FpAH1r) primers are shown in Table S4. Expression of *FpAH1* in the two transformants used was ∼3–5-fold higher than the wild type strain during *in vitro* culture and undetectable in the mutant as expected.

### Growth Rate Determination

A microtitre plate assay to monitor growth was performed based on the method described by Schmelz [74]. Briefly, each well contained 200 µL of basal media [72] with 5 mM glutamine as the nitrogen source with a final spore concentration of 1×10^4^ spores mL^−1^. Absorbance at 405 nm was measured using an iEMS microplate reader. For the *FpAH1* mutant test the plate remained in the instrument for the duration of the assay at ambient temperature. In the case of the *FpDLH1* mutant assay after an initial reading the plate was incubated at room temperature and readings commenced 22 hours post inoculation.

### *Fusarium* Crown Rot Virulence Assays

Assessment of fungal virulence during FCR was carried out using the method described previously [36]. For virulence testing of *FpAH1* mutant and complemented strains, isolates were inoculated onto 14 cm Synthetischer Nährstoffarmer Agar plates from potato dextrose agar plugs stored as water cultures and allowed to grow for 14 days under 12/12 hour day/night cycle under white and black fluorescent light at room temperature (∼22°C). Spores were harvested by flooding the plates with water and adjusted to the same concentration. To test the role of *FpDLH1* in virulence, spores were produced in shaking 1 L flasks containing 100 mL of 25% Campbell's V8 juice inoculated with a single plug taken from a 20% V8 juice plate. Flasks were incubated at room temperature for 8 days and spores harvested by filtration through miracloth followed by centrifugation (5,500×g) to remove media. Spores were resuspended in sterile water, counted and adjusted to the same concentration. Seeds were plated out two days prior to inoculation on wet paper towel in a 14 cm Petri dish and allowed to germinate on the laboratory bench. Germinated seeds were transferred to a 50 mL falcon tube containing 2 mL of the spore suspension and rolled gently to coat the seeds. Six to ten seeds were placed in a single paper towel, rolled up and tapped closed and placed in a jar containing water. Paper towel rolls were kept moist throughout the experiment. For the analysis of *FpAH1* mutants a concentration of 1×10^5^ spores mL^−1^ was used and plants were maintained on a laboratory bench without supplementary lighting. To test complemented *FpAH1* mutants and *FpDLH1* mutants, 4×10^5^ spores mL^−1^ were used and plants were maintained in a closed bench top biohazard cabinet with a cool white fluorescent light suspended from the roof of the cabinet (approximately 15 cm from the plants) providing 16 hours of light per day. Assays were scored by recording the number of plants that were alive as a proportion of the total plants.

### *Fusarium* Root-Rot Virulence Assays

Fungal strains were plated from PDA plug water storage onto 20% Campbell's V8 juice 1.2% agar plate and incubated for seven days at room temperature under white and black fluorescent light on a 12/12 hour day/night cycle. Seed for the assay were surface sterilized by soaking for 5 minutes in a 0.64% sodium hypochlorite-10% ethanol solution, followed by several rinses in sterile distilled water. Seed were plated onto three sheets of pre-wetted Whatmann no 3 12.5 cm filter disks in a 14 cm Petri dish. Seeds were incubated in the dark for 5 days at 4°C prior to germination at 20°C also in the dark. Germinated seedlings were distributed to Petri plates assembled with three filter papers wetted with 20 mL of sterile water prior to inoculation. Each plate contained 15–16 seedlings and was used for inoculation with one isolate. Inoculum consisted of agar plugs taken using either an inverted 1-mL pipette tip or a number 4 (6 mm) cork borer from the edge of the growing V8 agar plate. Plugs were placed on a single root per seedling about 1 cm below the seed with the mycelia in direct contact with the root. Plates were sealed with sealing film (PhytoTechnology Laboratories), incubated in a Thermoline illuminated incubator for 6 days at 20°C with 12 hours of light provided by fluorescent lamps. Assays were scored by measuring the length of the shoot.

### *Phaeosphaeria nodorum* Virulence Assays

Parental and mutant strains of *P. nodorum* were tested on the susceptible wheat cultivar Amery as previously described [75]. Latent period sporulation was assessed using detached leaves as described previously [76].

### *In planta* Expression Assays

Gene expression during infection of wheat and barley by *F. pseudograminearum* was performed using both a detached leaf infection assay and a root infection time course. For the latter, plants were inoculated as described for the root-rot virulence assay and root segments were harvested into liquid nitrogen at 24, 48 and 96 hours post inoculation. For expression in detached leaves, fourth leaf segments (7–8 cm long) of both hosts were taken from glasshouse-grown plants and each end of the cut leaf was sandwiched between water agar in a 14-cm Petri plate. Leaf segments were pierced at two points in a central region using a 200 µL pipette tip. A spore suspension of the wild type isolate (1×10^6^ sp mL^−1^) was placed on the wound sites and the plate was sealed with sealing film. Plates were maintained in a fluorescent bulb lit growth chamber (Thermoline) at 20°C with 12 hours of light. The whole leaf segment was harvested for RNA extraction. RNA extraction was performed using TRIzol reagent (Invitrogen) according to the manufacturer's instructions. Relative expression was compared to *β-tubulin*. Primers used for gene expression analysis are shown in Table S4. qRT-PCR was performed as previously described [73].

## Supporting Information

Dataset S1 **BLASTmatrix analysis of** ***F. pseudograminearum*** **proteins against 27 fungal species.** The BLASTmatrix tab contains the reciprocal best hit information in each of the 27 genomes for all 12448 *F. pseudograminearum* proteins. The identifier, percentage identity, match length, e-value and bit score are given for each of the hits. The FpSpecific156 tab contains the locus identifiers for all 156 *F. pseudograminearum* proteins that had no reciprocal best BLASTp hits in any of the 27 species. The FilteredBLASTmatrix239proteins tab contains the cereal pathogen specific set of 239 proteins and the bit score information for the matches across the 27 organisms.
(XLSX)

Figure S1 **Phylogenetic analysis of FPSE_07765 homologues in two other fusaria and bacteria.** The next best match in any fungi in GenBank (*Trichophyton verrucosum*) has been used as an out group. Fungal sequences are highlighted in grey boxes. Numbers on branches indicate approximate likelihood ratio test branch support values.
(EPS)

Figure S2 **Phylogram of FPSE_11233 orthologues in fungal and bacterial species.** No additional fungal matches were identified in GenBank other than those shown here at the e-value cut off of e^−5^. Fungal sequences are highlighted in a grey box. Numbers on branches indicate approximate likelihood ratio test branch support values.
(EPS)

Figure S3 **Slot blot hybridization of the** ***FpAH1*** **gene sequence to six** ***F. pseudograminearum*** **isolates (CS3096, CS3220, CS3270, CS3427, CS3487, and CS5834), five other fusaria (** ***F. graminearum*** **Ph1 and CS3005,** ***F. equiseti*** **CS3069,** ***F. acuminatum*** **CS5907 and** ***F. culmorum*** **CS7071), and** ***P. nodorum*** **(isolate SN15).**
(TIF)

Figure S4 **Expression of** ***FpAH1, FpDLH1 and FpAMD1*** **during infection of roots (A, C and E) and detached leaves (B, D and F) of wheat and barley.** For root infection assays, N = 4 with each replicate consisting of 15 pooled roots and detached leaf assays N = 3 with each replicate consisting of a single leaf segment.
(EPS)

Figure S5 ***AH1*** **gene knockouts in** ***Fusarium pseudograminearum*** **and** ***Phaeosphaeria nodorum*** **.** (A) Targeting construct (top) and wild type genomic locus (bottom) for disruption of *FpAH1*. 635 bp of the 5′ end of *FpAH1* was replaced by the neomycin phosphotransferase cassette driven by the *Aspergillus nidulans gpdA* promoter. (B) PCR screen to detect successful homologous recombination and gene deletion. PCR was performed with three primers (AHKOscr1, AHKOscr2 and gpdAr) as indicated in part A. Absence of the smaller wild type band and presence of the larger targeting vector specific band indicates successful knockout. (C) Growth of wild type and one *FpAH1* mutant in defined media. Error bars represent the standard error of the mean for three biological replicates. (D) Targeting construct (top) and wild type genomic locus (bottom) for disruption of *PnAH1* in *P. nodorum*.
(TIF)

Figure S6 **Virulence assay of the** ***Fusarium pseudograminearum*** **amidohydrolase 1 (** ***FpAH1*** **) mutants compared to the parental strain (CS3096) towards wheat 25 days post inoculation.** (A) survival of plants in the assay 25 days post inoculation. N = 3 with each replicate consisting of three or four paper towel rolls each with eight plants maintained in separate vessels. (B) Representative rolls of plants from the assay FCR assay (cultivar 2–49).
(TIF)

Figure S7 ***Phaeosphaeria nodorum*** **virulence assay with** ***PnAH1*** **mutants.** (A) Disease scores in leaf blight assays (B) Sporulation of mutants and wild type (SN15) after seven days of infection.
(EPS)

Figure S8 **Phylogenetic analysis of the dienelactone hydrolase (A) and amidase (B) proteins.** Sequences were selected based on reciprocal best blast hits in the BLASTmatrix analysis to (A) FPSE_08131 and FpDLH1 and (B) to FpAMD1. Numbers on branches indicate approximate likelihood ratio test branch support values.
(EPS)

Figure S9 ***Fusarium pseudograminearum*** **dienelactone hydrolase 1 (** ***FpDLH1*** **) knockout strategy and locus.** (A) Targeting construct (top) and wild type genomic locus (bottom) for disruption of *FpDLH1*. 891 bp of the *FpDLH1* gene was replaced by the neomycin phosphotransferase cassette driven by the *gpdA* promoter. (B) PCR screen to detect successful homologous recombination and gene deletion. PCR was performed using three primers (DLHKOscr1, DLHKOscr2 and gpdAr2) as indicated in part A. The lower band indicates the presence of the wild type locus and the absence of this band and the presence of the upper band is indicative of successful targeting. (C) Growth of the mutant and parental strain are indistinguishable in culture. Error bars represent the standard error of the mean for six biological replicates.
(EPS)

Figure S10 **Virulence assay of the** ***Fusarium pseudograminearum*** **dienelactone hydrolase 1 mutant (ΔFpDLH1) mutant towards barley cultivar Gairdner in crown rot (A and B) and root-rot (C and D) assays.** For A and B, N = 3 with each biological replicate consisting of four paper towel rolls consisting of 5–6 plants per roll. For C and D N = 15–16 plants. Error bars represent the standard error of the mean. Letters indicate statistically significant differences at P<0.05.
(TIF)

Table S1 **Numerical summary of BLASTmatrix comparative analysis of** ***F. pseudograminearum*** **proteins against those of 27 other fungi.**
(DOCX)

Table S2 **Putative orthologous amidohydrolase encoded in three** ***Fusarium*** **genomes.** Numbers in parentheses are percentage similarities at the amino acid level to the *F. pseudograminearum* sequence.
(DOCX)

Table S3 **Isolates used in this study and the database accession numbers of sequences from these isolates either generated here or accessed from previous publications.**
(DOCX)

Table S4 **Primers used in this study.** Lowercase letters indicate regions of homology for use in lambda phage mediated recombination or overlap PCR. Underlined sequences indicate restriction endonuclease target sites for use in cloning.
(DOCX)
