# Supplementary figures and images for: Comparative Pathogenomics Reveals Horizontally Acquired Novel Virulence Genes in Fungi Infecting Cereal Hosts
Source: PLoS Pathog. 2012 Sep 27;8(9):e1002952. doi: 10.1371/journal.ppat.1002952 (PMC3460631; doi:10.1371/journal.ppat.1002952)

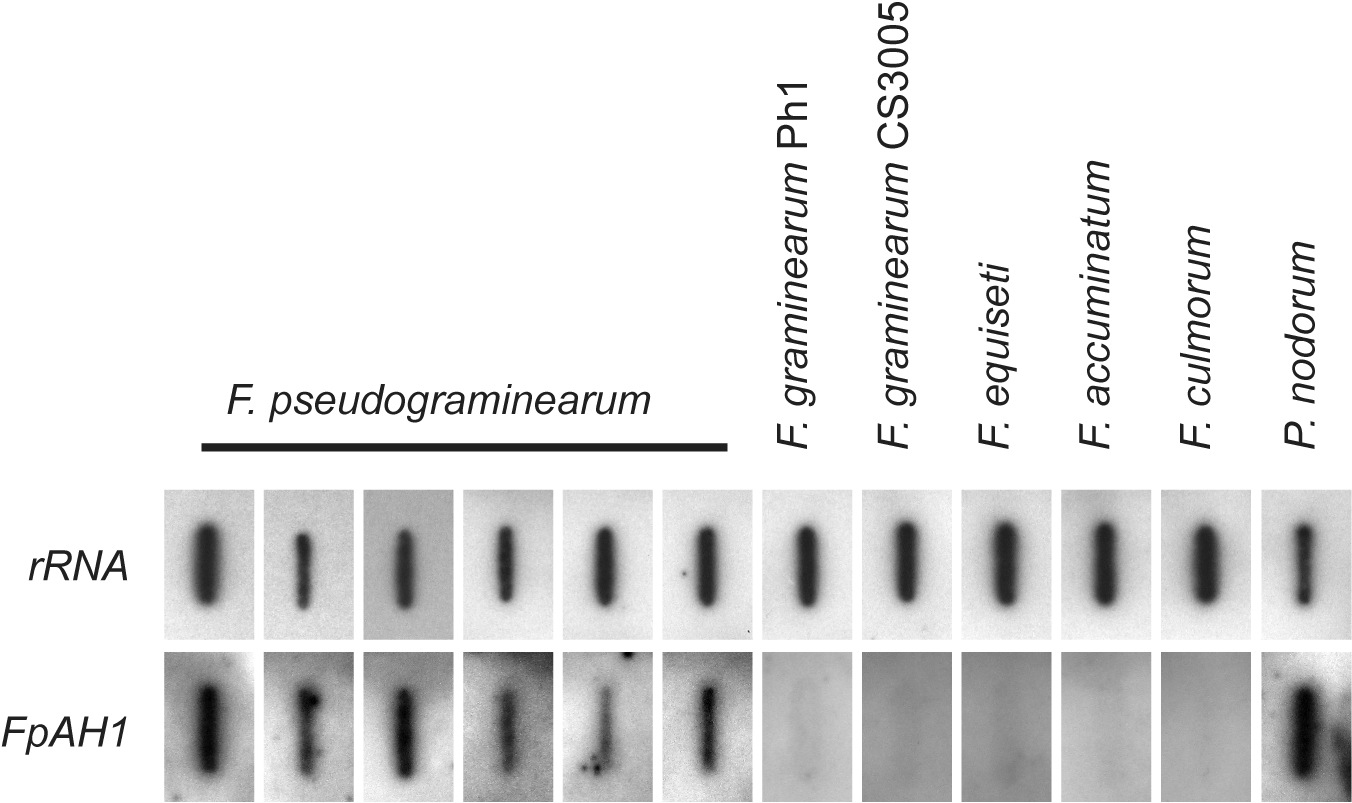

Supplement: Figure S3 — Slot blot hybridization of the FpAH1 gene sequence to six F. pseudograminearum isolates (CS3096, CS3220, CS3270, CS3427, CS3487, and CS5834), five other fusaria ( F. graminearum Ph1 and CS3005, F. equiseti CS3069, F. acuminatum CS5907 and F. culmorum CS7071), and P. nodorum (isolate SN15). (TIF) [file ppat.1002952.s004.tif]

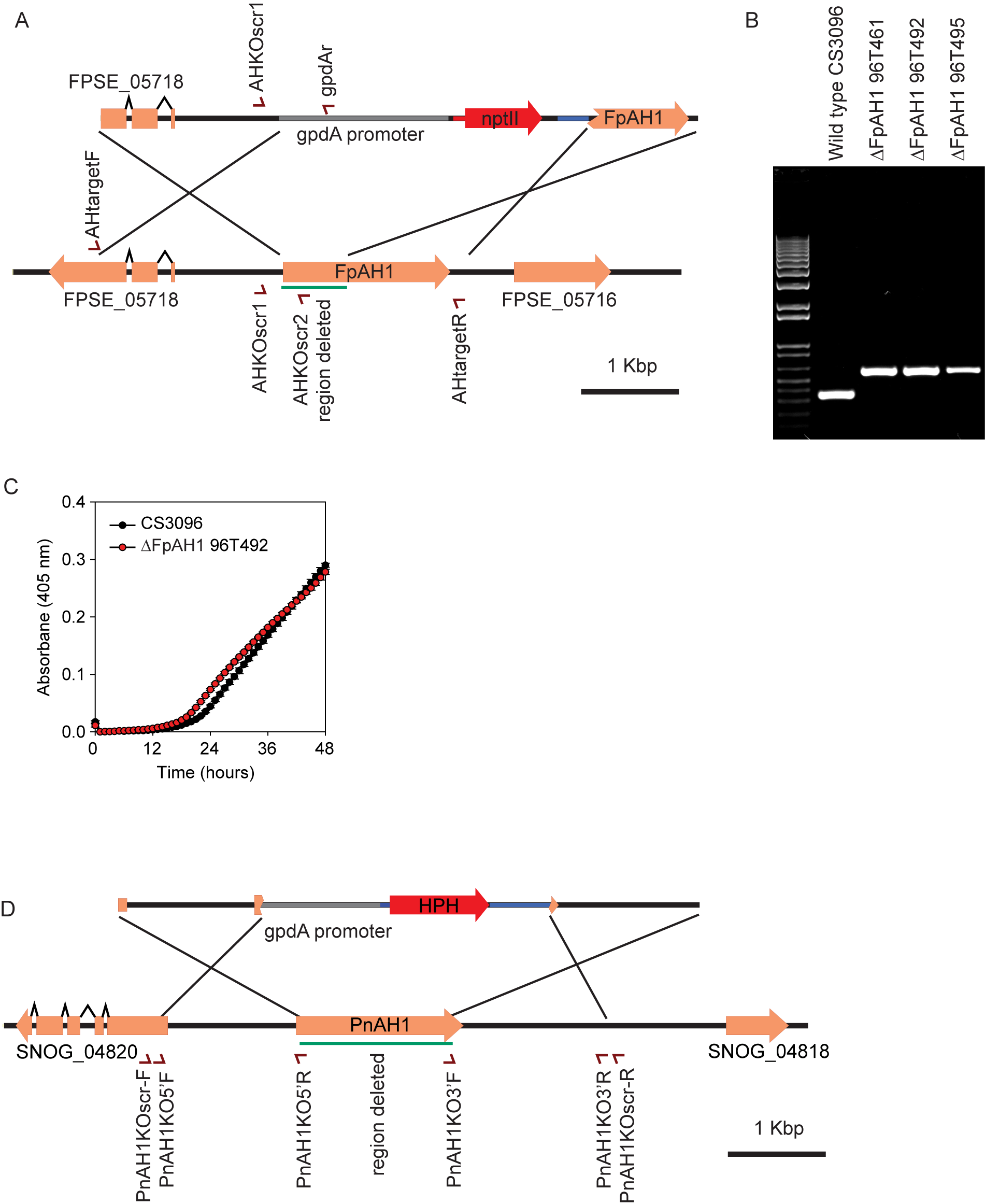

Supplement: Figure S5 — AH1 gene knockouts in Fusarium pseudograminearum and Phaeosphaeria nodorum . (A) Targeting construct (top) and wild type genomic locus (bottom) for disruption of FpAH1. 635 bp of the 5′ end of FpAH1 was replaced by the neomycin phosphotransferase cassette driven by the Aspergillus nidulans gpdA promoter. (B) PCR screen to detect successful homologous recombination and gene deletion. PCR was performed with three primers (AHKOscr1, AHKOscr2 and gpdAr) as indicated in part A. Absence of the smaller wild type band and presence of the larger targeting vector specific band indicates successful knockout. (C) Growth of wild type and one FpAH1 mutant in defined media. Error bars represent the standard error of the mean for three biological replicates. (D) Targeting construct (top) and wild type genomic locus (bottom) for disruption of PnAH1 in P. nodorum. (TIF) [file ppat.1002952.s006.tif]

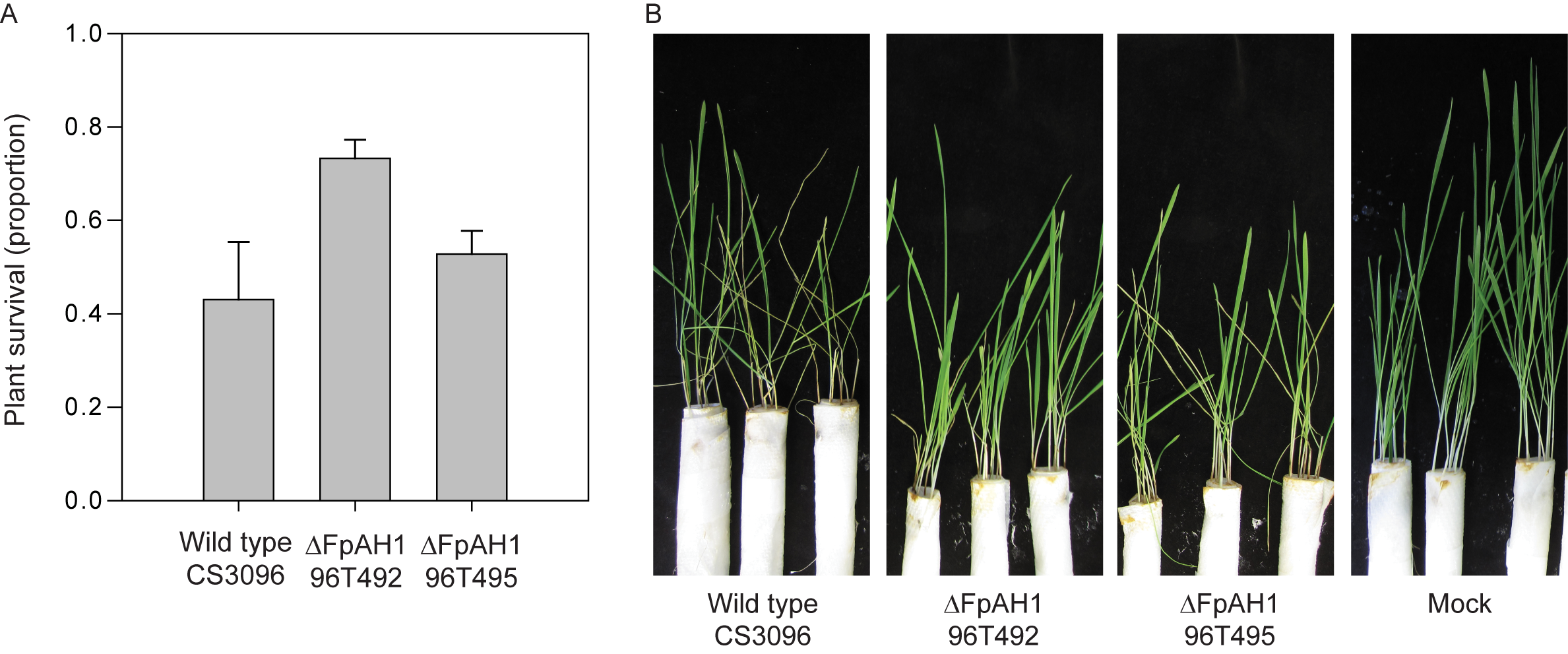

Supplement: Figure S6 — Virulence assay of the Fusarium pseudograminearum amidohydrolase 1 ( FpAH1 ) mutants compared to the parental strain (CS3096) towards wheat 25 days post inoculation. (A) survival of plants in the assay 25 days post inoculation. N = 3 with each replicate consisting of three or four paper towel rolls each with eight plants maintained in separate vessels. (B) Representative rolls of plants from the assay FCR assay (cultivar 2–49). (TIF) [file ppat.1002952.s007.tif]

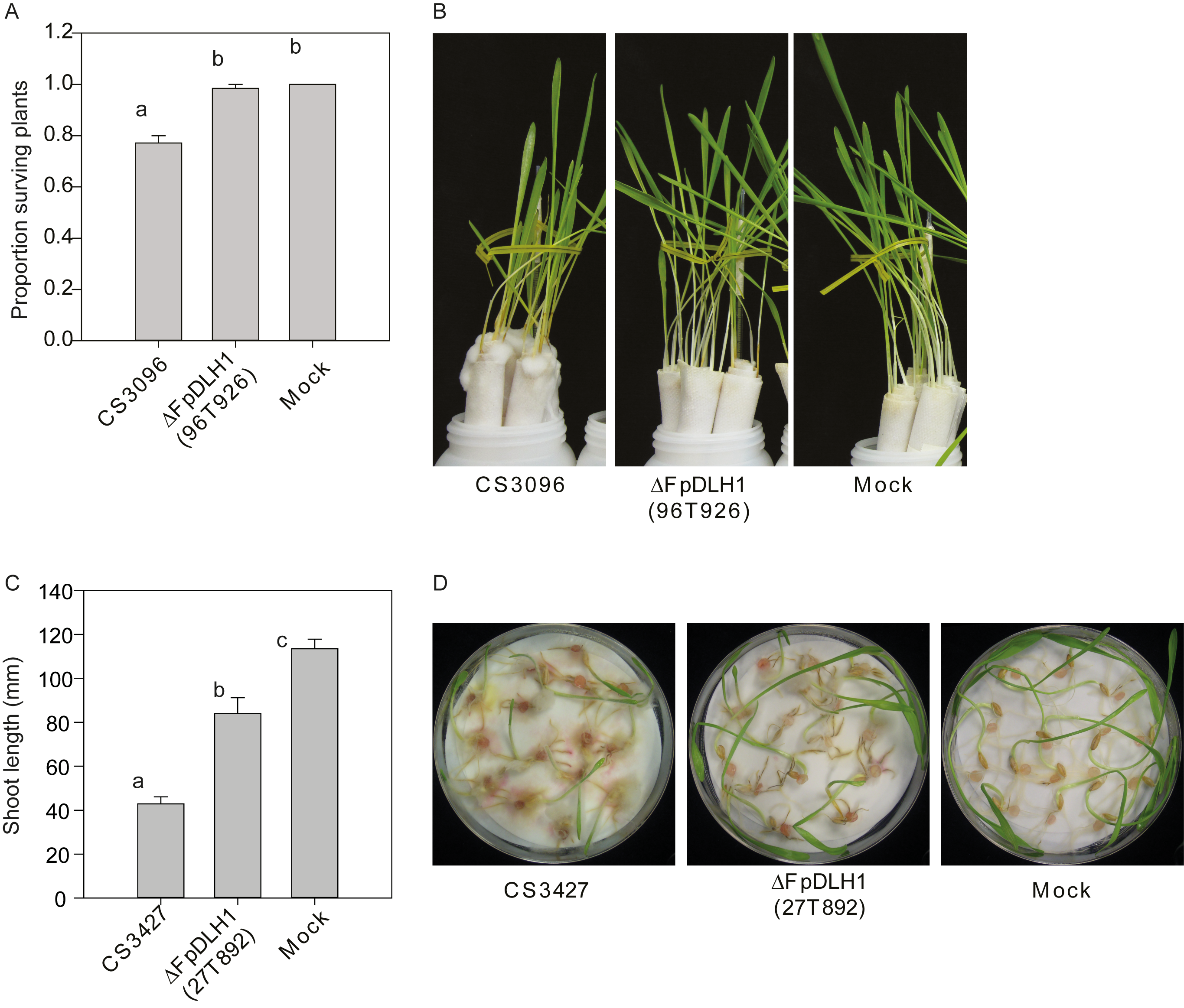

Supplement: Figure S10 — Virulence assay of the Fusarium pseudograminearum dienelactone hydrolase 1 mutant (ΔFpDLH1) mutant towards barley cultivar Gairdner in crown rot (A and B) and root-rot (C and D) assays. For A and B, N = 3 with each biological replicate consisting of four paper towel rolls consisting of 5–6 plants per roll. For C and D N = 15–16 plants. Error bars represent the standard error of the mean. Letters indicate statistically significant differences at P<0.05. (TIF) [file ppat.1002952.s011.tif]
