# Supplementary material for: Comparative Pathogenomics Reveals Horizontally Acquired Novel Virulence Genes in Fungi Infecting Cereal Hosts
Source: PLoS Pathog. 2012 Sep 27;8(9):e1002952. doi: 10.1371/journal.ppat.1002952 (PMC3460631; doi:10.1371/journal.ppat.1002952)
Supplement: Table S1 — Numerical summary of BLASTmatrix comparative analysis of F. pseudograminearum proteins against those of 27 other fungi. (DOCX) [file ppat.1002952.s012.docx]

Table S1: Numerical summary of BLASTmatrix comparative analysis of *F. pseudograminearum* proteins against those of 27 other fungi.

| **Category** | | | | **Gene count** | | | **Gene count with bacterial match ***(>200 bit score)** |
| --- | --- | --- | --- | --- | --- | --- | --- |
| *Fusarium pseudograminearum* specific | | | | 156 |  |  | 3 |
| Other cereal Pathogen specific* | | | | 239 |  |  | 17 |
|  | Fusarium specific | | |  | 214 |  | 11 |
|  |  | | *F. pseudograminearum* and *F. graminearum* |  |  | 203 | 8 |
|  |  | | *F. pseudograminearum* and *F. verticillioides* |  |  | 1 |  |
|  |  | | *F. pseudograminearum*, *F. graminearum* and *F. verticillioides* |  |  | 10 | 3 |
|  | Matches outside fusaria** | | |  | 25 |  | 6 |
|  |  | | *F. pseudograminearum* and only non-fusaria |  |  | 5 | 2 |
|  |  | | *F. pseudograminearum*, *F. graminearum* and other non-fusaria |  |  | 15 | 2 |
|  |  | | *F. pseudograminearum, F. verticillioides* and other non-fusaria |  |  | 1 |  |
|  |  | | *F. pseudograminearum, F. graminearum, F. verticillioides* and other non-fusaria |  |  | 4 | 2 |
| Non-cereal pathogen exclusive matches | | | | 32 |  |  |  |
|  | | *Fusarium* lineage matches | |  | 24 |  |  |
|  | | *F. pseudograminearum* and only non-fusaria | |  | 8 |  |  |

*A BIT score of >200 was required in at least one pathogen other than *F. pseudograminearum* for inclusion in this category

**Genes in other cereal pathogens included those also with a BIT score cut off of >100

***>200 bit score was used for bacterial matches
