# Supplementary material for: Comparative Pathogenomics Reveals Horizontally Acquired Novel Virulence Genes in Fungi Infecting Cereal Hosts
Source: PLoS Pathog. 2012 Sep 27;8(9):e1002952. doi: 10.1371/journal.ppat.1002952 (PMC3460631; doi:10.1371/journal.ppat.1002952)
Supplement: Table S2 — Putative orthologous amidohydrolase encoded in three Fusarium genomes. Numbers in parentheses are percentage similarities at the amino acid level to the F. pseudograminearum sequence. (DOCX) [file ppat.1002952.s013.docx]

Table S2: Putative orthologous amidohydrolase encoded in three *Fusarium* genomes. Numbers in parentheses are percentage similarities at the amino acid level to the *F. pseudograminearum* sequence.

| ***F. pseudograminearum* gene** | ***F. graminearum* orthologue** | ***F. verticillioides* orthologue** | ***F. oxysporum* orthologue** | ***Fungal hits of greater score than best bacterial hit*** |
| --- | --- | --- | --- | --- |
| FpAH1 | - | - | - |  |
| FPSE_00474 | FGSG_10082 (98) | FVEG_02691 (86) | FOXG_05447 (93) | 32 |
| FPSE_00725 | FGSG_10599 (97) | - | - | 1 |
| FPSE_02365 | FGSG_13458 (99) | FVEG_07691 (96) | FOXG_04527 (96) | 65 |
| FPSE_03227 | FGSG_05798 (97) | FVEG_04410 (90) | FOXG_07479 (89) | 65 |
| FPSE_05738 | FGSG_01993 (99) | FVEG_07448 (87) | FOXG_04321 (89) | 7 |
| FPSE_11444 | FGSG_01713 (99) | FVEG_09813 (94) | FOXG_10988 (94) | 68 |
| - | - | FVEG_05699 | - |  |
| - | - | FVEG_13869 | FOXG_13542 |  |

*BLASTp performed against NCBI’s nr
