# Supplementary material for: Comparative Pathogenomics Reveals Horizontally Acquired Novel Virulence Genes in Fungi Infecting Cereal Hosts
Source: PLoS Pathog. 2012 Sep 27;8(9):e1002952. doi: 10.1371/journal.ppat.1002952 (PMC3460631; doi:10.1371/journal.ppat.1002952)
Supplement: Table S3 — Isolates used in this study and the database accession numbers of sequences from these isolates either generated here or accessed from previous publications. (DOCX) [file ppat.1002952.s014.docx]

Table S3: Isolates used in this study and the database accession numbers of sequences from these isolates either generated here or accessed from previous publications.

| **Isolate** | **Species** | **Reference** | **Accession AH1** | **Accession EF1α** |
| --- | --- | --- | --- | --- |
| 10902001 | *F. pseudograminearum* | [1] | JN380863 | DQ382156 |
| 10902002 | *F. pseudograminearum* | [1] | JN380864 | DQ382157 |
| CS3096 | *F. pseudograminearum* | [3] | JN380865 | GU324917 |
| CS3220 | *F. pseudograminearum* |  | JN380866 | JN541051 |
| CS3270 | *F. pseudograminearum* | [1] | JN380867 | DQ382121 |
| CS3427 | *F. pseudograminearum* |  | JN380868 | JN541052 |
| CS3438 | *F. pseudograminearum* |  | JN380869 | JN541053 |
| CS3487 | *F. pseudograminearum* |  | JN380870 | JN541054 |
| CAN42 | *F. pseudograminearum* | [1] | JN380871 | DQ382149 |
| CAN43 | *F. pseudograminearum* | [1] | JN380872 | DQ382150 |
| CAN50 | *F. pseudograminearum* | [1] | JN380873 | DQ382154 |
| CS3002 | *F. pseudograminearum* | [1] | JN380874 | DQ382109 |
| CS3043 | *F. pseudograminearum* | [1] | JN380875 | DQ382110 |
| CS3064 | *F. pseudograminearum* | [1] | JN380876 | DQ382112 |
| CS3074 | *F. pseudograminearum* | [1] | JN380877 | DQ382114 |
| CS3454 | *F. pseudograminearum* | [1] | JN380878 | DQ382129 |
| CS3507 | *F. pseudograminearum* | [1] | JN380879 | DQ382130 |
| CS3616 | *F. pseudograminearum* | [1] | JN380880 | DQ382132 |
| CS3685 | *F. pseudograminearum* | [1] | JN380881 | DQ382135 |
| CS3891 | *F. pseudograminearum* | [1] | JN380882 | DQ382138 |
| CS3894 | *F. pseudograminearum* | [1] | JN380883 | DQ382141 |
| CS3900 | *F. pseudograminearum* | [1] | JN380884 | DQ382144 |
| CS3911 | *F. pseudograminearum* | [1] | JN380885 | DQ382147 |
| CS5639 | *F. pseudograminearum* |  | JN380886 | JN541055 |
| CS5791 | *F. pseudograminearum* |  | JN380887 | JN541056 |
| CS5894 | *F. pseudograminearum* |  | JN380888 | JN541057 |
| CS5963 | *F. pseudograminearum* |  | JN380889 | JN541059 |
| NRRL28062 | *F. pseudograminearum* | [2] | JN380890 | AF212468 |
| NZ67 | *F. pseudograminearum* | [1] | JN380891 | DQ382161 |
| NZ69 | *F. pseudograminearum* | [1] | JN380892 | DQ382162 |
| TUR006 | *F. pseudograminearum* | [1] | JN380893 | DQ382163 |
| TUR047 | *F. pseudograminearum* |  | JN380895 | JN541062 |
| CS5907 | *F. acuminatum* |  | na | JN541058 |
| TUR057 | *F. cerealis* |  | na | JN541063 |
| CS7071 | *F. culmorum* |  | na | JN541060 |
| CS3069 | *F. equiseti* |  | na | JN541050 |
| CS3005 | *F. graminearum* | [3] | na | GU370497 |
| Ph1 | *F. graminearum* |  | na | 2789790* |
| TUR020 | *F. graminearum* |  | na | JN541061 |
| SN15 | *P. nodorum* |  | 5972107* |  |
| J30 | *P. avenaria* f. sp. *tritici 1* |  | JX079697 |  |
| J38 | *P. avenaria* f. sp. *tritici 1* |  | JX079698 |  |
| IR10_6.2b | *P. avenaria* f. sp. *tritici 1* |  | JX079699 |  |
| I34 | *P. avenaria* f. sp. *tritici 3* |  | JX079700 |  |
| I35 | *P. avenaria* f. sp. *tritici 3* |  | JX079701 |  |
| I36 | *P. avenaria* f. sp. *tritici 3* |  | JX079702 |  |
| I37 | *P. avenaria* f. sp. *tritici 3* |  | JX079703 |  |
| J48 | *P. nodorum* |  | JX079704 |  |
| J39 | *P. nodorum* |  | JX079705 |  |
| H47 | *P. nodorum* |  | JX079706 |  |
| H25 | *P. nodorum* |  | JX079707 |  |
| IR10_2.1c | *P. nodorum* |  | JX079708 |  |
| H15 | *P. nodorum* |  | JX079709 |  |
| IR10_7.3b | *P. avenaria* f. sp. *tritici 1* |  | JX079710 |  |
| Chi01_54a | *P. nodorum* |  | JX079711 |  |
| IR10_3.1b | *P. avenaria* f. sp. *tritici 1* |  | JX079712 |  |
| IR05_C5.1b | *P. avenaria* f. sp. *tritici 1* |  | JX079713 |  |
| IR05_1.2a | *P. avenaria* f. sp. *tritici 1* |  | JX079714 |  |
| IR10_2.1b | *P. avenaria* f. sp. *tritici 1* |  | JX079715 |  |
| IR10_6.2a | *P. avenaria* f. sp. *tritici 1* |  | JX079716 |  |
| IR10_5.2a | *P. avenaria* f. sp. *tritici 1* |  | JX079717 |  |
| IR10_7.3c | *P. avenaria* f. sp. *tritici 1* |  | JX079718 |  |
| IR10_9.1b | *P. avenaria* f. sp. *tritici 1* |  | JX079719 |  |
| IR10_9.1c | *P. avenaria* f. sp. *tritici 1* |  | JX079720 |  |
| IR10_9.2c | *P. avenaria* f. sp. *tritici 1* |  | JX079721 |  |
| IR10_9.2a | *P. avenaria* f. sp. *tritici 1* |  | JX079722 |  |
| IR10_9.2b | *P. avenaria* f. sp. *tritici 1* |  | JX079723 |  |
| ARKW2 | *P. nodorum* |  | JX079724 |  |
| ARKW5 | *P. nodorum* |  | JX079725 |  |
| ARKW3 | *P. nodorum* |  | JX079726 |  |
| 07SAReb_Cf1_9a | *P. nodorum* |  | JX079727 |  |
| 07SAReb_Cf1_5a | *P. nodorum* |  | JX079728 |  |
| 07SAReb_Cf1_13b | *P. nodorum* |  | JX079729 |  |
| CH01A2.17 | *P. nodorum* |  | JX079730 |  |
| 07SAReb_Cf1_1a | *P. nodorum* |  | JX079731 |  |
| Aus01-F10 | *P. nodorum* |  | JX079732 |  |
| Aus01-F8 | *P. nodorum* |  | JX079733 |  |
| CH01A2.9 | *P. nodorum* |  | JX079734 |  |
| ARKW1 | *P. nodorum* |  | JX079735 |  |
| SA95.38 | *P. nodorum* |  | JX079736 |  |
| IR10_9.1a | *P. nodorum* |  | JX079737 |  |
| SA95.21 | *P. nodorum* |  | JX079738 |  |
| Chi0155a | *P. nodorum* |  | JX079739 |  |
| Chi0154b | *P. nodorum* |  | JX079740 |  |
| IR10_9.3c | *P. nodorum* |  | JX079741 |  |
| SA95.25 | *P. nodorum* |  | JX079742 |  |
| IR10_9.3b | *P. nodorum* |  | JX079743 |  |
| IR10_2.1a | *P. nodorum* |  | JX079744 |  |
| H7.1a | *P. nodorum* |  | JX079745 |  |
| IR10_11.2a | *P. nodorum* |  | JX079746 |  |
| IR05_H4.1a | *P1* |  | na |  |
| IR05_A1 3.1a | *P2* |  | na |  |
| IR05_H6.2b | *P2* |  | na |  |
| IR05_C4.3a | *P1* |  | na |  |

* GenBank gene identifiers shown for Ph1 *EF1α* and SN15 *PnAH1* sequences

1. Scott JB, Chakraborty S (2006) Multilocus sequence analysis of *Fusarium pseudograminearum* reveals a single phylogenetic species. Mycol Res 110: 1413-1425.

2. O'Donnell K, Kistler HC, Tacke BK, Casper HH (2000) Gene genealogies reveal global phylogeographic structure and reproductive isolation among lineages of *Fusarium graminearum*, the fungus causing wheat scab. Proc Natl Acad Sci USA 97: 7905-7910.

3. Obanor F, Erginbas-Orakci G, Tunali B, Nicol JM, Chakraborty S (2010) *Fusarium culmorum* is a single phylogenetic species based on multilocus sequence analysis. Fungal Biol 114: 753-765.
