# Supplementary material for: Comparative Pathogenomics Reveals Horizontally Acquired Novel Virulence Genes in Fungi Infecting Cereal Hosts
Source: PLoS Pathog. 2012 Sep 27;8(9):e1002952. doi: 10.1371/journal.ppat.1002952 (PMC3460631; doi:10.1371/journal.ppat.1002952)
Supplement: Table S4 — Primers used in this study. Lowercase letters indicate regions of homology for use in lambda phage mediated recombination or overlap PCR. Underlined sequences indicate restriction endonuclease target sites for use in cloning. (DOCX) [file ppat.1002952.s015.docx]

Table S4: Primers used in this study. Lowercase letters indicate regions of homology for use in lambda phage mediated recombination or overlap PCR. Underlined sequences indicate restriction endonuclease target sites for use in cloning.

| **Primer** | **Sequence** | **Use** |
| --- | --- | --- |
| AHtargetF | GCAAGCCTGTCAATGTCTCA | KO construction |
| AHtargetR | AAAAATTTCGCCACGAATTG |  |
| AHNeoF | ccttcgagatttagcccgttctcatctctgcctgtcatttatcccgaatcAGAAGAACTGGAGGGGTGGT | KO construction |
| AHNeoR | tctctggcgtgatgaggggtggcatcttcttgactatggaagcccaatgcTAACCTGAGGCTATGG | KO construction |
| AHKOscr1 | TTTACGAGAGGCCCAACCTA | Mutant screening 355 bp product in wild type |
| AHKOscr2 | CCAGCCACCGATATGTTCTT |  |
| gpdAr | ACGTTTGACTTGCATTGTGC | Mutant screening 600 bp product with AHKOscr1 |
| DLHtargetF | ACGACCGAGGGTATCTGTTG | KO construct primers |
| DLHtargetR | GCTCCGTACCAGACACCAAT |  |
| DLHNeoF | ccaacccagccttacccagttgccgaatcattcctgagcaagccaaaagtACGGCGTAACCAAAAGTCAC | KO construction |
| DLHNeoR | ccacaggaagcatcatccagattgaaggagtcgatacatacgtcgcCAAGCTTTAACCTGAGGCTATG | KO construction |
| DLHKOscr1 | GCCCAACTTCACAGGACAAT | Mutant screening 444 bp product in wild type |
| DLHKOscr2 | AATGAGGCTCGCACAAGAGT |  |
| gpdAr2 | GAGCTCACGAGTTCGTCACA | Mutant screening 644 bp product with DLHKOscr1 |
| FpAHDiversityF | GATCCATGGGGCAATATCAC | Diversity analysis and *FpAH1* probe synthesis |
| FpAHDiversityR | GTTGATCCAAGTGGCAGGTAA |  |
| PnAHDiversityF | TGATCTGTGATTTTGCTGCTC |  |
| EF1αf | ATGGGTAAGGARGACAAGAC | Confirmation of speciation of *Fusarium* isolates [1] |
| EF1αr | GGARGTACCAGTSATCATG |  |
| PnAH1KO5'F | GTGGCGTAGGTGTTTCCA | KO construction |
| PnAH1KO5'R | acttttggttacgccgtctGCGTCGGAATAGACAATGTC |  |
| PnAH1KO3'F | atgcatggttgcctagtgaCTGGAAAGAATGCCGACTT |  |
| PnAHKO3'R | ATGAGGTTTTCTAGGCCGAG |  |
| PnAH1KOscr-F | ATTTGGCGCTATCCGAAC | Mutant screening |
| PnAH1KOscr-R | CATTGAACAAAGCCCCATC |  |
| β-tubulinF | GTCATTACACCGAGGGTGCT | qPCR |
| β-tubulinR | CCACCAAGAGAGTGGGTGAT |  |
| FpAH1f | CGCTATCGGCTCTAATGAGG | qPCR designed in region deleted in FpAH1 mutants |
| FpAH1r | CAAACCTGGGGTTATGGTTG |  |
| FpAH1f2 | TCGCCGGAGATACACCTTAC | qPCR |
| FpAH1r2 | TTCCAGGCGAGCTTGTAACT |  |
| AH-OXf | AGCTATCGATATGAACGTCGAACAAGTT | Clone *FpAH1* behind TrpC promoter for complementation of mutants |
| AH-OXr | AGCTGAATTCTGCTGGCAGATAGTTAAG |  |
| ITS1-F | CTTGGTCATTTAGAGGAAGTAA | rRNA probe synthesis [2] |
| ITS4 | TCCTCCGCTTATTGATATGC |  |

1. O'Donnell K, Kistler HC, Cigelnik E, Ploetz RC (1998) Multiple evolutionary origins of the fungus causing Panama disease of banana: concordant evidence from nuclear and mitochondrial gene genealogies. Proc Natl Acad Sci USA 95: 2044-2049.

2. Gardes M, Bruns TD (1993) ITS primers with enhanced specificity for basidiomycetes - application to the identification of mycorrhizae and rusts. Mol Ecol 2: 113-118.
